# Bio-Nanocarriers for Lung Cancer Management: Befriending the Barriers

**DOI:** 10.1007/s40820-021-00630-6

**Published:** 2021-06-12

**Authors:** Shruti Rawal, Mayur Patel

**Affiliations:** Department of Pharmaceutics, Institute of Pharmacy, Nirma University, SG Highway, Chharodi, Ahmedabad, Gujarat 382 481 India

**Keywords:** Biomimetic nanoparticles, Exosomes, Circulating tumor cells, Mesenchymal stem cells, Theranostics

## Abstract

Lung cancer is a complex thoracic malignancy developing consequential to aberrations in a myriad of molecular and biomolecular signaling pathways. It is one of the most lethal forms of cancers accounting to almost 1.8 million new annual incidences, bearing overall mortality to incidence ratio of 0.87. The dismal prognostic scenario at advanced stages of the disease and metastatic/resistant tumor cell populations stresses the requisite of advanced translational interdisciplinary interventions such as bionanotechnology. This review article deliberates insights and apprehensions on the recent prologue of nanobioengineering and bionanotechnology as an approach for the clinical management of lung cancer. The role of nanobioengineered (bio-nano) tools like bio-nanocarriers and nanobiodevices in secondary prophylaxis, diagnosis, therapeutics, and theranostics for lung cancer management has been discussed. Bioengineered, bioinspired, and biomimetic bio-nanotools of considerate translational value have been reviewed. Perspectives on existent oncostrategies, their critical comparison with bio-nanocarriers, and issues hampering their clinical bench side to bed transformation have also been summarized.

## Highlights

- Due to their multifaceted oncological applications and immense translational potential, the bio-nanocarriers and nano-biodevices are being conceived as a futuristic panacea for cancer.
- Aspects impeding promising prognosis of lung cancer, various nano-biotools, and their plausible benefits over the conventional nanocarriers for lung cancer management have been briefed upon in this review.
- Research findings from relevant investigations, perspectives, and stipulations for the overall management of lung cancer have also been deliberated.

## Graphical abstract

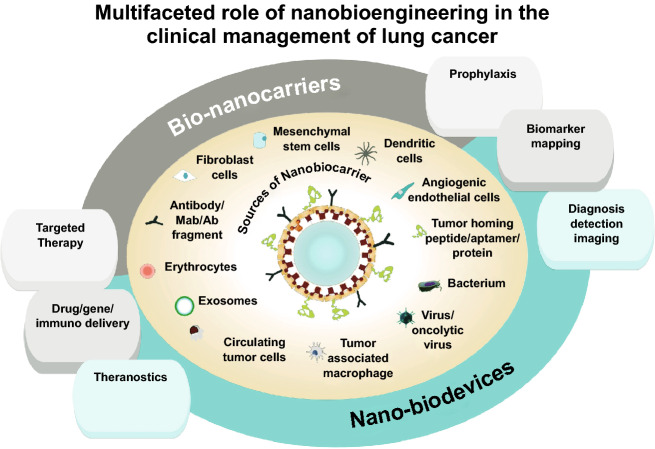

## Introduction

The global burden of cancer is rising insidiously. With disquietingly high mortality rates (1 in 5 cancer deaths worldwide), lung cancer has been entitled as one of the most lethal forms of cancer [1]. As per cancer statistics provided by the American Cancer Society, 11.6% of all new cancer incidences and 18.4% of cancer mortalities (1.8 million in 2018) are ascribed to lung cancer [2]. Lung cancer is a highly heterogenic disease with complex clinical repercussions and a poor prognosis. The major reproachful factors that worsen the prognosis of lung cancer are lack of prophylactic modality, diagnosis at an advanced stage, extremely mutable tumor milieu, diverse genetic and epigenetic aberrations, multi-drug resistance, and metastatic dissemination [3].

Nanotechnology has provided a platform to design nanocarriers and nanodevices that manifest their activity through various magnetic, thermal, electrical, and optical properties by the virtue of their tunable composition, shapes, structure, and sizes (1–100 nm at least in one dimension) [4]. Nanotechnology has leveraged the precincts of biomedical and oncology science beyond many physiological barriers for the effective management of complex diseases like cancer. However, the sluggish approval pace and poor clinical performance of the nano-medicines implore the scientific community to foster breakthrough innovation in clinical and translational oncology for achieving better prognosis in lung cancer patients.

On the other hand, concurrent efforts in the fields of molecular biology, omics, genetic engineering, and cancer biology have revolutionized biotechnology and bioengineering to a significant extent. Sophisticated biotechnology tools have extensively contributed to oncology over recent years toward gathering information about complex cancer biological milieu and biomolecular signaling thereof. However, despite tremendous advances in both of these individual scientific disciplines, the gap between bio-sciences and nanotechnology has undermined their actual potential. The realization of the need to unify these individual disciplines to gain miraculous biomedical outcomes has surfaced up with nanobioengineering. Nanobioengineering is amongst the most challenging domain of bionanotechnology that is advancing rapidly. Nanobioengineering is an applied science that integrates the fundamentals of basic sciences like physics, chemistry, and biology to engineer material properties in nano-dimensions for manifold bio-medical and medical applications. It has assisted contrive novel nanobiotools and strategies for comprehending, managing, and revolutionizing clinical aspects of lung cancer management.

Despite the advances in oncological sciences, clinical management of lung cancer is still a perplexing task. The present article scrutinizes abstruseness in the current scenario of lung cancer management and discusses nanobioengineering strategies to overcome the same. A comprehensive overview of the bio-nanotools (bio-nanocarriers and nano-biodevices) for multifaceted applications in lung cancer along with a comparative assessment of the principal advantages and shortcomings has been briefed upon. In the clinical context, disease management comprises four (quadripartite) different aspects: prophylaxis/prevention, diagnosis, treatment/therapeutics, and therapeutic drug monitoring (facilitated through theranostics). A critical appraisal of the research works relating to the application of bio-nanotools for each of these aspects of lung cancer management has been elaborated.

Nanobioengineering has assisted the development of nano-based cancer vaccines and chemopreventive nanomedicine that can be employed for secondary prophylaxis in lung cancer. Novel bio-nanotools like nano-bio-based contrast and imaging agents, nanoprobes, nanobiosensors, biomarker detection devices, and high-throughput nanodevices have been researched to reinforce early-stage diagnostic techniques for detection, imaging, and molecular imaging at high detection speeds with high specificity and sensitivity. Additionally, the advances in the ‘nano-bioera’ in oncotherapy have resulted in a paradigm shift from conventionally non-targeted therapies to tumor-targeted therapies. Nano-bioengineering has extended its application to radiotherapy, immunotherapy, phototherapy, gene therapy, and combination therapy as well as novel therapies to facilitate multimodal oncotargeting of lung cancer. Nanobioengineering had a late advent in the therapeutic segment of oncotargeting due to delayed evidence of translational and clinical incompetency of a multitude of nanocarrier-based therapeutics reported in the literature. The search for competent targeting strategies has led to the development of novel third-generation nanocarriers termed ‘nano-biocarriers’*.* Nano-biocarriers are bioengineered, bioinspired, biogenic, bio-mimicking, and bio-hybrid nanocarriers, cells, or vesicles that employ biological moiety/biomimetic moiety/bioinspired/bioderived moiety as a bioactive or as active targeting vector/ligand. Unlike the other nanocarriers, the nano-biocarriers have distinct advantages like being multi-targeted, highly bio-interactive, biocompatible, intrinsically camouflaged, and scaleable. Additional attributes like tropism and host integration may also aid in personalized therapy, or in enhancing the efficiency and safety of bio-nanocarriers.

The multi-functional bio-nanotools have extended their application in theranostics as well. Some researchers have also proposed multi-functional nanoparticles to merge all the facets like diagnosis, therapy as well as prevention with the use of a single nano-modality. Therapeutic drug monitoring, real-time monitoring, and image-guided therapy are some major applications that may be facilitated with the help of theranostics. The contemporary erudition and prospects pertinent to the subject have been summarized as an endnote.

## Challenges Associated with Current Lung Cancer Management

Despite the advances in lung cancer diagnostics and therapeutics, lung cancer is still an incurable disease. As stated earlier, metastatic, malignant, resistant, or advanced stage lung cancers are difficult to treat within the precincts of currently available knowledge of lung cancer biology. Chemoprevention and cancer vaccine development have been subject to oncological research for decades, but have not been substantiated through clinical appraisal. While there has been considerable advancement in imaging and screening techniques, the current detection techniques fail to diagnose the early stage of the disease due to a lack of biomarker detection at low concentrations. The other limitation of the prevalent diagnostic option is the lack of identification of the molecular/genetic variant of the tumor sub-type due to high inter-patient diversity. Techniques to preclude the aforementioned disadvantages while facilitating the identification of novel biomarkers and molecular targets are being sought. While considering the therapeutic aspects, combination chemotherapy, nanocarrier-based therapy, biological therapy, and immunotherapy have clinically materialized as adjuvant and neoadjuvant therapy to surgery and radiotherapy. With the advent of novel targeted biological agents and immunotherapeutic agents for the treatment of non-small cell lung cancer (NSCLC), there has been a considerable improvement in therapeutic outcomes & patient survival rates [5]. However, the treatment scenario of other subtypes of lung cancer such as small cell lung cancer (SCLC) and malignant pleural mesothelioma is disquieting and contradictory. Striking difference in the treatment protocols for each sub-type of lung cancer can be attributed to the underlying cytological, histological, genetic, epigenetic and molecular target diversity. A comprehensive understanding of underlying molecular signaling and targets is obligatory for enhancing the therapeutic and prognostic efficiency in all clinical cases of lung cancer. Diverse receptor upregulation indicates involvement of different molecular signaling pathways and molecular targets in the pathogenesis of different lung cancer sub-types. A brief summary of receptors identified in different sub-types of lung cancer is presented in Table 1 [6].

**Table 1:** Lung cancer types, sub-types and featured receptors

Lung cancer type	Sub-type	Featured upregulated receptors
Non-small cell lung cancer (NSCLC) (80–85%)	*Adenocarcinoma* Major form of NSCLC orginating from mucus-secreting cells of peripheral lung tissue with slow progression *Squamous cell carcinoma* Originates from airway lining of pleural cells *Large cell carcinoma *Cancer originating from central part od the lungs, often of neuroendocrine origin, aggressive and advanced at diagnosis	Epidermal growth factor receptor (EGFR), human epidermal growth factor receptor 2 (HER2/neu), folate receptor alpha (FRα), TNF-related apoptosis-inducing ligand (TRAIL) death receptors DR4 & DR5, tetraspanins CD151, mesenchymal–epithelial transition (MET), estrogen receptors (ERα and ERβ), G-protein-coupled estrogen receptors, progesterone receptors (PRA and PRB), peroxisome proliferator-activated receptor gamma (PPARγ), somatostatin, neuropilins receptor proteins (NRP), echinoderm microtubule-associated protein like 4 anaplastic lymphoma kinase (EML4-ALK), vascular endothelial growth factor receptor (VEGFR), cluster differentiation 44 (CD44), bombesin receptors (BBR1, BBR2, and BBR3), oxytocin and vasopressin receptors
SCLC (10–15%)	Smoking is the major cause, is neuroendocrine in origin, often non-resectable, metastatic and with high relapse rates	Epidermal growth factor receptor (EGFR), somatostatin, CD24, bombesin receptors (BBR1, BBR2, and BBR3), bradykinin receptors, oxytocin and vasopressin receptors, insulin-like growth factor 1 receptor, fibroblast growth factor receptors
Others (~ 5%)	Pleural mesothelioma Lung carcinoid tumors Hamartomas Adenoid cystic carcinoma Adenoid cystic lymphoma Adenoid cystic sarcoma	Epidermal growth factor receptor (EGFR), folate receptor alpha (FRα), cluster differentiation 44 (CD44), interleukin 4 receptor alpha, estrogen receptor (ERβ)

As demonstrated in Table 2, there are very few targeted therapeutic options approved by the FDA for the treatment of SCLCStill worse, there is only one therapy (the combination of the NovoTTF-100L system with platinum-based chemotherapy and pemetrexed) that has been approved by the FDA for the treatment of malignant pleural mesothelioma [7]. Lack of knowledge about molecular targets in lung cancer variants except for the non-small cell lung carcinoma (NSCLC), lack of bio-stability of therapeutic and targeting moieties, failure of enhanced permeation retention (EPR) phenomenon, and active targeting in complex biological milieu may be the liable factors. A closer understanding of cellular, genetic, and molecular alterations in the emergence, progression, and metastasis of lung carcinoma are imperative for promoting the identification of novel targets. The concept of therapeutic drug monitoring and theranostics is still at a preliminary stage of their development and demands extensive research. In this context, seeking a resort of bionanotechnology for prophylaxis, diagnosis, therapeutic, and theranostics of lung cancer may prove to be a boon to mankind.

**Table 2:** Drugs approved for the treatment of lung cancer

Chemotherapeutic agents	Biologically targeted therapeutic agents	Immunotherapeutic agents
*Drugs approved for the treatment of small-cell lung carcinoma*		
Doxorubicin	Everolimus	Atezolizumab
Etoposide phosphate		Pembrolizumab
Methotrexate		Nivolumab
Mechlorethamine		
Topotecan		
*Drugs approved for the treatment of non-small cell lung carcinoma*		
Albumin-stabilized nanoparticle formulation of paclitaxel (Abraxane®)	Afatinib dimaleate	Atezolizumab
Carboplatin	Alectinib	Bevacizumab
Carboplatin + paclitaxel	Brigatinib	Durvalumab
Docetaxel	Ceritinib	Necitumumab
Doxorubicin	Crizotinib	Nivolumab
Gemcitabine	Dacomitinib	Pembrolizumab
Mechlorethamine	Entrectinib	Ramucirumab
Methotrexate	Erlotinib.HCl	
Paclitaxel	Everolimus	
Pemetrexed disodium	Gefitinib	
Vinorelbine Tartrate	Osimertinib Mesylate	
	Trametinib	

## Nanobioengineering and Bio-nanotools: Types and Sub-types

**Fig. 1:**
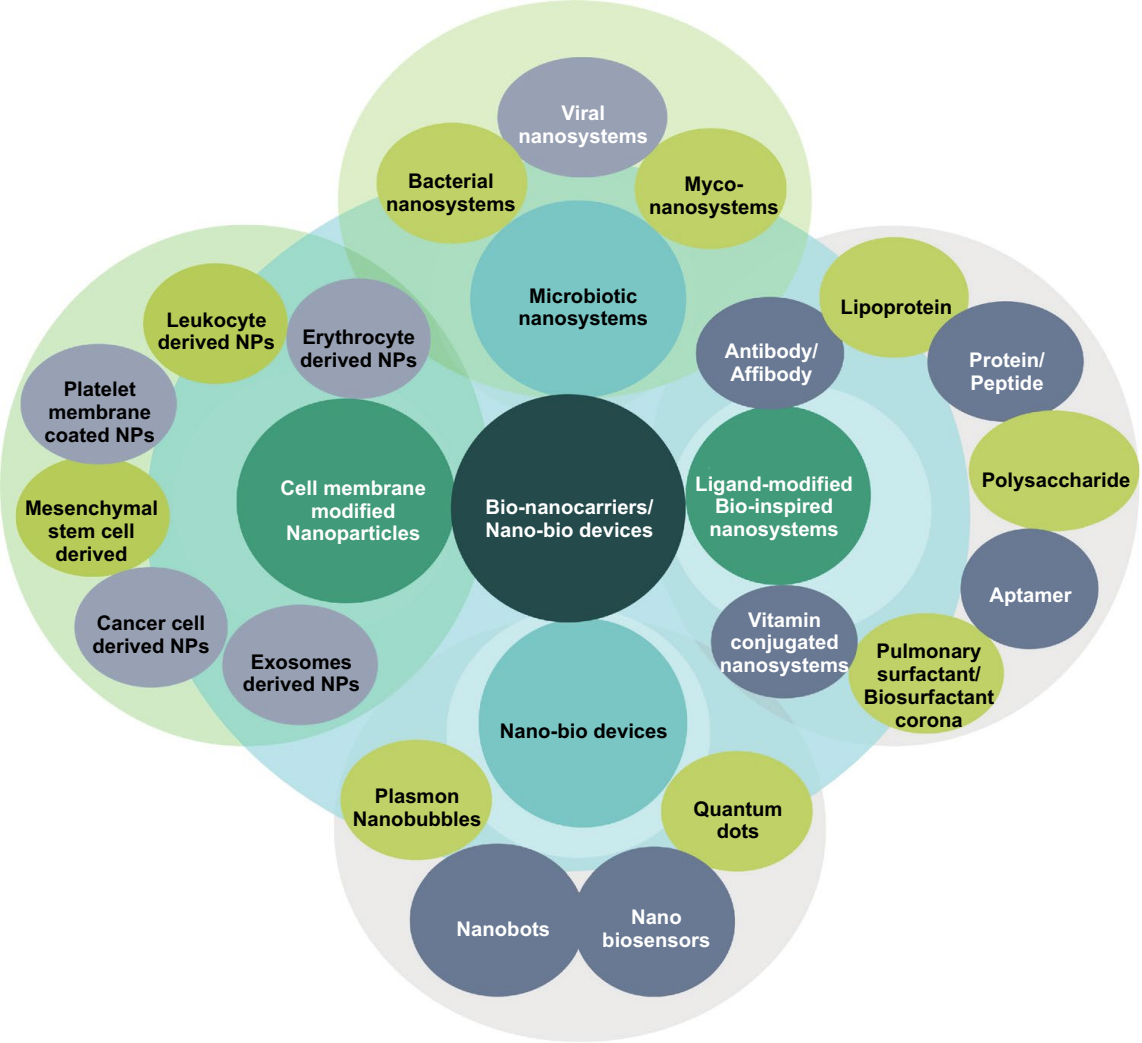
Bio-nanocarriers and nano-bio-devices for oncological applications

Various bio-nanotechnology-derived tools like nano-biocarriers and nano-bio-devices have recently gained tremendous scientific interest in addressing issues of lung cancer management. The most widely explored bio-nanocarriers for oncological applications can be summarized as: (1) microbiotic nanosystems and bio-nanocarriers, (2) cells and cell membrane-derived nanosystems, (3) ligand-conjugated nanosystems, and (4) nano-bio-devices (Fig. 1).

### Microbiotic Nanosystems and Bio-nanocarriers

Amidst significant controversies, microbiotic-based nanosystems are progressively gathering compelling preclinical and clinical evidences in their favor for being applicable in oncology [8–14]. Because the micro-organisms like bacteria, viruses, and fungi have a strong innate tropism toward specific cells/organs, their vectorization potential seems promising. Also, they bear an immense potential for cell-specific delivery to the tumors due to higher intracellular penetration and cell uptake. Advances in biomics and bioengineering techniques have significantly reduced the pathogenicity and biotoxicity of the microbiome tools while enhancing their vectorization potential. There have been several ongoing efforts in the utilization of these microbiome-derived tools like oncolytic viruses (virotherapy) or oncolytic bacteria for addressing various issues pertaining to tumor targeting [15]. Various microbiotic bio-nanocarriers that have been researched for the management of various malignancies including lung cancer have been discussed in the following sections:

#### Bacterial Bio-nanocarriers

Bacterial properties like high motility, immune evasion ability, chemotaxis, invasion capability, cytotoxicity, tumor vasculature accumulation, and abundance and composition of pathogen-associated molecular patterns (PAMP) are the major factors that have upraised the interest of researches worldwide for tumor targeting [16]. With the help of several bioengineering tools, novel bacterial systems with attenuated pathogenicity, high tumor-targeting ability, strategic drug expression, and versatile payload deliverability are being researched and developed [16, 17]. Some of the most investigated bacteria for oncological applications are Streptococcus, Salmonella, Proteus, Listeria, Clostridium, Bifidobacterium, Escherichia, Caulobacter [18].

A promising scenario can be predicted from the number of ongoing clinical trials for the bacterial treatment of cancer [16]. Bionanotechnology advances have come up with several bacterial-based nanoplatforms for oncological applications. Various types of bacterial bio-nanocarriers like bioengineered bacteria, bacterial minicells, bacterial membrane-derived nanovesicles (proteoliposomes), bacterial ghosts, bacterial components like the “S-layer”, bacterial cell membrane, bacterial derivatives like the endospores (spore vaccine), magnetosomes, and bacterially derived polymers (e.g., alginate, hyaluronic acid, cellulose, poly-l-lactic acid, and Ɛ-poly-l-lysine) have been reported to be employed in oncology [19]. Nanobio-hybrids are fabricated by employing physical attachments, chemical propagation, and biological reconstruction/engineering methods.

##### Bioengineered Bacteria

Bioengineered bacteria are bacteria that are genetically modified for specialized oncological applications. Various tumor-targeted strategies like the release of anticancer agents (cytotoxic agents, cytokines, antigens, and antibodies), genetic transfer (cytotoxic and anti-angiogenic agents, cytokines and growth factors, tumor antigens, gene silencing (shRNA), gene triggering strategies (signal promoters), combinations with other treatments (anti-vascular agents, chemotherapeutic drugs, heat shock proteins, heavy metals), radiation and imaging strategies like bioluminescence, fluorescence, magnetic resonance (MRI), positron emission (PET), etc., have been reported [18].

##### Bacterial Minicells

These are enucleated spherical derivatives of ~ 400 nm diameter formed as a result of polar division from the bioengineered bacteria [20]. Bacterial minicells are known to target tumors due to the interaction of bispecific antibodies with O-polysaccharide moiety of the minicell and tumor cell-specific receptors. Erbitux® EDVs_PAC_, developed by EnGeneIc, is a prototype of such targeted minicells, undergoing clinical trials for the treatment of various solid tumors. The minicells were reported to be loaded with chemotherapeutic agents like doxorubicin (DOX), cisplatin, and paclitaxel (PTX) and gene-based therapeutics like siRNA, plasmid DNA encoding the short hairpin RNA (shRNA) [9, 10]. Different minicell formulations like the ^HER2^minicells_DOX,_^EGFR^minicells_PAC,_ and ^EGFR^minicells_DOX_ were reported to have high antitumor efficacy in several tumor xenograft models including lung cancer [8]. Besides this, the bacterial minicells have been reported to be bio-stable for a long duration (~ 6 h) and prevent opsonization by macrophages [21].

##### S-Membrane-Derived Nanovesicles

The surface layer or S-layer of bacteria is the outermost membrane of the bacteria, constituting its envelope. These simple protein/glycoprotein structures offer an enormous possibility of manipulations to formulate various bio-nanocarriers through bioengineering. Some of the most attractive features of the S-layer that have been exploited to meet various oncological applications are highly porous structural scaffold (~ 30–70% porosity), regularly repeating sub-units with different shapes and geometry, highly modifiable protein/glycoprotein structure, and self-assembling ability. Details about the use of S-layer for various oncological aspects such as diagnostics, sensors, cancer vaccine, bio-marker, and nano-targeted drug delivery have been elaborately explained elsewhere [19]. The S-layer has been reported to serve as a targeting ligand for the targeting of various nanocarrier systems like liposomes and emulsomes [22, 23]. Additionally, the S-layer can serve as a carrier for antigen, and vaccine delivery is being researched upon for its use as a cancer vaccine and in immunotherapy of various forms of cancers [24]. Application of such S-layer-coated bio-nanocarrier-based biosensors and detection techniques have also been well reported [22, 25].

##### Magnetosomes

‘Magnetosensitive’/magnetotactic bacteria or the bacteria that respond to the external magnetic field were first observed by Salvatore Bellini in 1963 [26]. It has been reported that the subcellular organelles of bacterial species like Magnetospirillumm bavaricum and Magneticum contain magnetic-iron-containing minerals such as greigite (Fe_3_S_4_) and magnetite (Fe_3_O_4_), referred to as magnetosomes. The biomedical application of magnetic principles was commenced in 1960 by Freeman et al., following which there was a significant rise in this direction. While magnetic nanoparticles like superparamagnetic iron oxide (SPIONs) have gained significant attention from researchers worldwide due to their magnetic targetability, surface mouldability, low toxicity, and biocompatibility, the magnetosomes also present a promising alternative for similar applications [27]. The magnetosomes can be applied for targeted drug delivery of various oncological modalities like gene or theranostics [28–30]. Biomagnetites have also been employed for the development of biosensors to detect mutations [31, 32]. Several research works that have been utilized in the context of lung cancer management have been discussed later [31, 33–35].

##### Bacterial Ghosts

The envelope of gram-negative bacteria emptied of its cytoplasmic contents through cloned lysis/tunnel formation is termed as bacterial ghosts. Despite the removal of cytoplasmic contents, the bacterial ghosts conserve the cell morphology and cell surface structure. Their intrinsic adjuvant properties make them ideal for the formulation of vaccines or aid as immunotherapeutic. Besides this, the surface of these bionanocarriers can be modified to present native antigens or DNA and other recombinant antigens simultaneously to elicit specific humoral/cellular responses to target the cancer cells. The bacterial ghosts may contain several types of payloads like drugs, imaging agents, etc., to facilitate its applicability for diverse functions. Batch fermentation, product recovery, and tangential flow filtration can yield high loads of bacterial ghosts. To ensure the pathogenicity of the bacterium, they are subsequently purified with staphylococcal nuclease A and β-propiolactone. These bacterial bio-nanocarriers may be lyophilized to meet large-scale production needs for various biotechnological applications including lung cancer oncotheranostics [36–38].

##### Bacteria-Derived Outer Membrane Vesicles (Proteoliposomes) (OMVs)

OMVs of the bacteria are components of gram-negative bacteria, composed of latent membrane protein (LMP), phospholipids, lipoproteins, exogenous protein epitopes, flagellin, nucleic acids, and peptidoglycan, through which the bacteria communicate. The OMVs are derived from the bacteria during normal bacterial growth or under stressful growth conditions [19]. The OMVs elicit an immune response by interacting with Toll-like receptor 4 (TLR4) [39]. The OMVs are advantageous in terms of biostability of the cargo as it provides protection from the DNase, RNase, protease, and extreme pH [40]. The OMVs derived very small proteoliposomes have been reported to facilitate the targeted delivery of various therapeutic, imaging, and theranostic agents to lung cancer cells [11, 39, 41–43]. In addition to drug delivery applications, the OMVs are also applied for the development of cancer vaccines, immunotherapy, and very recently for photoacoustic imaging of tumors [44].

##### Bacterial Polymer-Based Nanocarriers

The first bacterial bio-polymer discovery was made by Louis Pasteur in 1861, which later became known as dextran. Various bacteria like Leuconostoc mesenteroides, Acetobacter xylinumin, and Ralstonia eutropha, Streptomycetaceae actinobacteria, B. subtilis, Salmonella, Sarcina, Achromobacter, Gluconacetobacter, Agrobacterium, Aerobacter, Rhizobium, Agrobacterium, and Azotobacter are known to synthesize and accumulate various biopolymers. Such bio-polymers include polysaccharides (hyaluronic acid, dextran, alginate, starch, K30 antigen, xanthan, and glycogen), polyamides (poly (γ-glutamic acid), polypeptides and proteins), polyesters (polythioesters and polyhydroxyalkanoates), polyphosphates and polyphenols such as lignin. Nanoparticles composed of or nanocarriers that are surface-modified using any of these bio-polymers have been employed in diverse applications like drug delivery, biosensor fabrication, etc. [45–52]. Applications of bacterial nano-bio-hybrids from preventive, detection, imaging, therapeutic, and theranostics in lung cancer are discussed in the forthcoming section.

#### Bioengineered Viruses, Bacteriophage, and Viral Bio-nanocarriers

Immense transgene expression capacity, tropism, and cell-specificity make the oncological application of viral vectors an attractive approach. However, due to the high pathogenicity and virulence of viruses, this strategy was highly discouraged [53]. However, advances in bioengineering and biotechnology have facilitated viral modifications to reduce their virulence and enhance the targeting ability. Bioengineered viruses like oncolytic viruses, bacteriophage, and viral bio-nanocarriers are recently being employed for the management of various cancers including lung cancer [53–55]. Viral bio-nanocarriers are specially fabricated by employing physical methods, chemical methods, and biotemplation methods. Due to the presence of surface functional groups like the amide, aniline, thiol, carboxyl and phenol moieties, chemical modifications in the viral groups can be easily done [14].

Viral bio-nanocarriers can be formulated by chemical methods like direct conjugation, wherein the nanomaterial and viral capsid are conjugated directly [56, 57]. Viral components can be bioconjugated over the surface of various nanocarriers like quantum dots, gold nanoparticles, bacteriophage, etc., by employing this method. The bioconjugation potential of viruses with chemotherapeutic agents, imaging agents, proteins, chromophores, and nanomaterials has also been reported to confer specificity and biostability to such cargoes [14]. Owing to the features like structural symmetry and chemical self-assembly of the viral capsids, the viral bio-nanocarriers, consisting of nanoparticles deposited over the viruses, may be constructed using the biotemplation method [58–60].

#### Fungal and Yeast Bio-nanocarriers

Oncological research toward the development of fungal and yeast-based bio-nanocarriers has recently gained interest amongst scientists worldwide [61, 62]. Fungus and yeast cells have been reported to serve as a targeting vector for various nanoparticle systems for diverse oncological applications [62]. Fungal bio-derived polymers like β-glucan, chitin, and chitosan have also been reported to facilitate the encapsulation of various oncological modalities [63, 64]. It has been reported that the Glucan biopolymer has potential therapeutic efficacy in lung cancer [65]. Bio-synthesized/biogenic silver nanoparticles and zinc oxide nanoparticles from various fungi and yeasts are also gaining significant attention for their anticancer applications [66–68]. Furthermore, bioengineered yeast-derived vacuoles have also been explored for oncological applications [69]. Fungal bio-nanocarriers that utilize the electrostatic method/chemical modification method for conjugation of nanoparticles to yeast cells or vice versa are other sub-types of bio-nanocarriers that have been formulated for oncological applications in lung cancer [70–72]. The fungal bio-nanocarriers and nano-biocarriers provide an excellent platform for reasons of being an easily modifiable, cost-effective, and safer alternative to the other microbiome-derived counterparts [61, 62].

### Cells and Cell Membrane-derived Bio-nanocarriers

Novel archetypes of drug delivery carriers and devices are being developed extensively to meet oncological challenges. The bioinspired and biomimetic nanoparticles are gaining significant attention amongst researchers worldwide owing to their distinct attributes like non-iatrogenicity, biocompatibility, biodegradability, and tailoring ability. Mammalian cell and cell-membrane-derived bio-nanocarriers are progressively being sought for devising novel targeting strategies for the treatment of various malignancies including lung cancer. Cell-based targeting oncovectors that have been researched include the whole cell-based carriers, cell membrane-derived nanocarriers, membrane-cloaked bio-nanocarriers, microvesicle-based nanocarriers, and exosome-based bio-nanocarriers. Some of the sources from which such bio-nanocarriers are formed include erythrocytes, leukocytes, platelets, mesenchymal stem cells, cancer cells, and exosomes. In addition to biomimetic camouflaging, the cell-based nano-biocarriers exhibit excellent biocompatibility, multi-molecular and intrinsic targeting ability, self-stealthing ability, and favorable host bio-integration for multiple oncological applications.

#### Erythrocyte-Derived Bio-nanocarriers

Amongst the nano-biocarriers, erythrocytes have gained significant attention from the scientific community due to additional advantages like large quantities of the cell membrane, high internal capacity volume (185–191 μm^3^), high biostability and biodurability, high in vitro storage ability, simplistic isolation methods, long systemic circulation (~ 120 days), high loading capacity and low aggregation. Erythrocytes, erythrocyte membrane-based bio-nanocarriers, and nanoerythrosomes are some of the most explored cell-based nano-biocarriers [73, 74].

Erythrocyte/red blood cell (RBC)-based drug delivery was pioneered by Gardos in 1953, who first attempted the loading of ATP inside the erythrocyte ghosts. Led by the premise of the work by Gardos, Marsden and Ostling reported the use of erythrocytes for carrying dextran in 1959. Thereafter, the term “carrier red blood cells” was coined in 1979 after a breakthrough was brought about by an erythrocyte-based delivery for delivery of β-glucosidase and β-galactosidase to treat Gaucher’s disease. Subsequently, the RBC-based drug delivery was attempted for drugs like gentamicin, leukemia, and L-asparagine-dependent leukemia [75].

The first nano-biohybrid of its kind was developed and reported by Lejeune et al. in 1994. The developed erythrocyte-derived liposomes termed “nanoerythrosomes” were formed by the physical squeezing of the RBC ghosts through the membranes of definite pore sizes. However, due to issues such as vesicle aggregation, lack of structural integrity, and rapid systemic clearance, such a system was not found to be applicable. Later on, in 2013, Zhang’s group developed novel toxin nanosponges that were composed of a polymeric core and erythrocyte shell, which revitalized the field with breakthrough opportunities that underlie the formulation approach. Erythrocyte membranes have several immune evading mechanisms mediated by CD47 receptor activation, CD59, homologous restriction protein (HRP), C8 binding protein (C8bp), membrane cofactor protein (MCP), decay-accelerating factor (DAF), and complement receptor1 (CR1) due to such a ‘self-recognizing’ [76, 77].

**Fig. 2:**
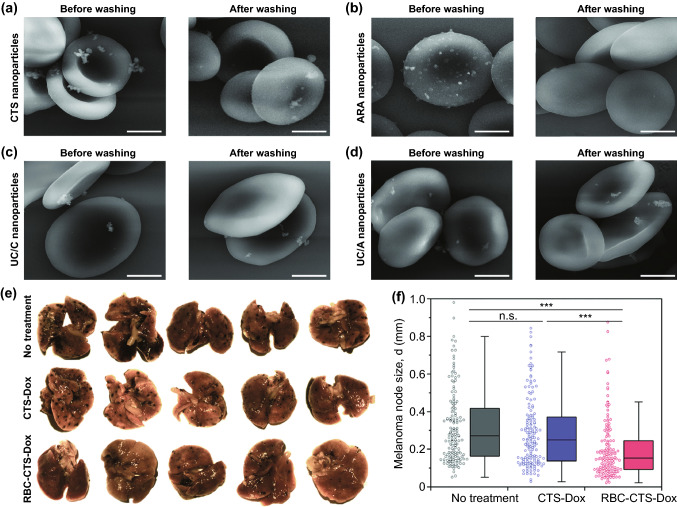
Electron microphotographs of erythrocyte complexes with **a** chitosan (CT), **b** glucuronic acid, **c** cationic non-coated, and **d** anionic non-coated 100 nm nanoparticles (scale = 2 μm) before and after washing. **e** Images of metastatic foci on lung tissues excised from untreated animals, animals treated with CT-doxorubicin nanoparticles and RBC bound-CT-doxorubicin nanoparticles in metastatic melanoma murine model **f** melanoma node size (mm^3^) (data representing 100 metastasis in each group, distinct values represented by Tukey-type box plots. Statistical implications determined using Welch’s t test (n.s,***P < 0.001*,*P < 0.01, and *P* > 0.05). Adapted from Ref. [86] with permission from Royal Society of Chemistry RSC

The circulation half-life of RBC membrane-coated nanoparticles was reported to be 2 times greater than the half-life of PEGylated nanoparticles [74, 78]. There are several methods employed for the preparation of the RBC-coated bio-nanocarriers. Physical methods like hypotonic dialysis, hypotonic dilution, hypotonic hemolysis, hypotonic pre-swelling, electrical breakdown, and osmotic pulse have been widely employed in the preparation of RBC-coated bio-nanocarriers. Some other methods that are employed are the chemical perturbation method and chemical perturbation method in combination with an electrical breakdown, lipid fusion, intrinsic cell uptake, and endocytosis [78–83]. Formulating erythrocyte-based nanocarriers have a distinct advantage over the natural erythrocytes due to a reduction in direct physicochemical interaction of payload with the membrane components, their consecutive leakage, and toxicity. Some interesting studies have exquisitely described how RBC hitchhiking [84] and other nano-biointeractions of RBCs (radiotherapy assistance, etc.) have been employed in context with lung cancer therapy (Fig. 2) [85–87].

#### Leukocyte (WBC)-Derived Bio-nanocarriers

Leukocytes or WBCs are major saviors of the immune system that participate in the majority of the morbidities in addition to cancer. The special attributes that attract the scientists to employ them as drug carriers are: (1) specialized transmigration capability to the cancer site, (2) tumor endothelial adhesion, and (3) chemotaxis-driven targeting ability [76]. However, due to low systemic circulation half-life (~ 20 days), their application in drug delivery is limited. To overcome the limitations of the natural leukocyte-based drug delivery, the leukocyte-based bio-nanocarriers are being researched. The leukocyte-derived bio-nanocarriers can be classified into the following sub-types:

##### Monocyte/Macrophage Bio-nanocarriers

Macrophages are modified monocytes that are major constituents of tumoral mass (~ 50% of tumor mass) and are vital regulators of the immune response. Tumoral chemotaxis of these immune cells is mediated by chemoattractants such as chemokine ligand 2 (CCL 2, 5, 7, 8, 22), CXCL 8 and 12, PDGF, VEGF, endothelial monocyte-activating polypeptide (EMAPII), and colony-stimulating factor-1 (CSF-1). Moreover, localization of the macrophages to the tumor stroma is driven by hypoxia, which results in the formation of tumor-associated macrophages (TAMs) [88]. Due to tumor tropism of such kind, macrophages and monocytes are attractive tumor-targeting vectors [88, 89]. Factors that impede the enhanced permeation of nanoparticles and therefore their intratumoral penetration, such as high tumor interstitial pressure, discontinuous tumor vasculature and dense tumor stroma, lead to decreased intratumoral bioavailability. However, bare loading of payload into macrophages/monocytes may result in drug inactivation and randomized drug release [90]. Formulating the bio-nanocarriers using macrophage/monocyte membrane may offer potential benefits over the conventionally used nanoparticles and drugs by facilitating intratumoral penetration and localization of diverse payloads such as polysaccharides, endotoxins, complement, lipoproteins, affibodies, and antibodies. Bio-nanocarriers based on macrophages have been reported with the drug crystals, liposomes, emulsions, bacteria, and gold particles [90].

##### Dendritic Cell-Derived Bio-nanocarriers

Dendritic cells (DC) are one of the most important immune system mediators that unify the innate and adaptive responses through the formation of major histocompatibility complexes to expose foreign antigens by the formation of antigen-presenting cells (APCs) [91]. The plasmacytoid DCs (pDCs) and the myeloid DC-based bio-nanocarriers are highly favored for immunotherapy and devising cancer vaccines. The approach of employing DCs as the vaccine is approved by the FDA for the treatment of metastatic hormone-refractory malignancies. In a clinical phase III trial of one such DC-based formulation, i.e., Provenge® (Sipuleucel-T), it was observed to increase the median survival rate of patients by four months. Due to the requirement of a low cell dose, a single DC formulation was claimed to deliver five doses. Due to the presence of mannose receptor, Fc receptor, complement receptor, C-type lectin receptor, and the DEC-2005 receptor, the Dcs have an immense immune-evoking capacity and cell interaction ability. These attributes of DCs present an opportunity to fabricate immunomodulatory nanoparticles. These DC-based bio-nanocarriers can facilitate cell uptake, intracellular activation, and APC presentation, mediated by MHC-I and MHC-II [88].

##### Neutrophil-Derived Bio-nanocarriers

Neutrophils are perceived as excellent bio-carriers due to the advantages like being the most populous sub-types of the leukocytes, excellent transmigration capacity across blood vessel endothelium toward cancer cells, and aggressive response to inflammation [92, 93]. The neutrophils can be localized to the tumor sites and form tumor-associated neutrophils (TANs), which offer an immense potential of forming the bio-nanocarriers.

##### Lymphocyte-Derived Bio-nanocarriers

Amongst the two main subtypes of lymphocytes, i.e., the T cells and the B cells, the T cells are the most explored for drug delivery applications. With the use of gene-editing techniques, the autologous T cells are bioengineered using chimeric antigen receptors (CAR) and employed as CAR-T therapy in cancer patients [94]. However, due to the loss in the cell viability of the T cells post-injection, bio-nanocarriers composed of the T-cell membrane were developed for the first time by Stephen et al., who proposed a novel means of attaching the nanoparticles with T cells, loaded with IL-5 and IL-21. It was observed to release the interleukins in an autocrine fashion from these carrier cells, leading to T cell-mediated immune response and tumor elimination.

#### Platelet-Derived Bio-nanocarriers

The interplay of platelet localization and tumor metastasis has long been exploited in the field of oncology. Altered RNA and ultrastructure of platelets have been associated with the diagnosis of some malignancies including lung cancer [95, 96]. Recently, bio-nanocarriers composed of platelet membrane-conjugated nanoparticulate systems (PNP) are being researched for their oncological applications. The PNP has been reported to exhibit enhanced collagen binding, enhanced endothelial adherence, and significantly greater antigen binding as compared to their conventional counterparts [97]. Some of the platelet-derived bio-nanocarriers employed in lung cancer therapy are discussed in reference [95, 97, 98].

#### Mesenchymal Cell (MSC)-Derived Bio-nanocarriers

Due to their explicit anti-inflammatory and immunomodulatory modes of action, more than 550 clinical trials are being executed employing the mesenchymal cells [99]. The MSCs have been reported to possess inflammation-driven tumor tropism mediated by the adhesion ligands like Sialyl Lewis X (SLeX) and P-selectin glycoprotein ligand (PSGL-1) as well as CXCL12, CXXR4, and CCR2. The presence of surface antigenic moieties and innate targeting ligands largely favor their use as a vector for oncotargeting. MSCs have been employed for the delivery of protein and peptide oncotherapeutics such as CX3CL1, interferon, and interleukins [100]. The bio-nanocarriers like MSC nanoghosts and MSC-derived nanovesicles are highly favored over the MSCs due to their safety in terms of non-tumorigenicity. The molecular mechanisms of tumor-targeting, the modes of genetic and non-genetic bioengineering of MSC-based bio-nanocarriers have been described elsewhere in detail [101].

#### Cancer Cell-Derived Bio-nanocarriers

Cancer cell membrane-modified bio-nanocarriers (CCMNs) are widely researched for oncological targeting of various payloads [102, 103]. CCMNs confer unique attributes to the nanoparticles like homotypic targetability, unique binding ability, and selective cell uptake to primary and metastatic tumor nodules [102, 104–107]. Their biomimetic camouflaging properties prevent their premature immune system clearance and prolong their systemic circulation. Additionally, due to the ease of culture of the cancer cells and their robustness, they serve as an extremely vital source for the mass production of the cancer cell membrane [108]. The CCMNs have been employed for phototherapy, targeted chemotherapy, enhanced tumor imaging, and molecular diagnosis in addition to their immunomodulatory effect [108].

#### Extracellular Vesicles and Exosome-Derived Nanosystems and Bio-nanocarriers

Diverse subtypes of membrane-coated particles that are formed by the mammalian cells for mediation of paracrine signaling are termed as ‘extracellular vesicles (EVs)’ [109]. EVs are classified into the following subtypes: microvesicles, nanovesicles, exomeres, exosomes, oncosomes, apoptotic bodies, and arrestin domain-containing protein 1-mediated microvesicles (ARMMs) [110]. These naturally existing biocarriers carry payloads such as lipids, proteins, nucleic acids, and other bioactive cargoes. The EVs have been postulated to have a major role in the bio-signaling interplay between the tumor cells to cause immunosuppression, tumor progression, and tumor metastasis. Due to their natural payload-carrying capacity, they have been explored for diverse oncological applications [111, 112].

Amongst EVs, exosomes are one of the most explored nanocarriers. The EVs of 30–150 nm diameter that are secreted by most of the body cells into the body fluids are termed exosomes [113]. Exosomes derived from the cancer cells and salivary exosomes have been reported to have plausible oncological applications [111, 114]. The exosomes originate from the inward endosomal budding and subsequent fusion of the cell membrane with multivesicular bodies (MVBs) containing the intraluminal vesicles (ILVs) [110]. The structural composition of exosomes depends upon the chemical composition of the parent cells from which they emerge. The exosomal surface overexpresses characteristic proteins, namely tetraspanins (TSPAN6, TSPAN 8, CD9, CD63, and CD81), cell adhesion proteins (lactadherin, integrin), cell-type-specific proteins (FasL, MHC-I, MHC-II, and WNT), and heat shock proteins (Hsc70), that directs the function, cell interaction, cell uptake, and the fate of exosomes [110, 115].

### Bioinspired/Bioderived Ligand Conjugated Nanosystems

Bio-nanocarriers in which the conventional nanosystems/nanoparticles are modified for facilitating tumor targeting by the means of natural, bioengineered, bioinspired, or bioderived biomolecules are discussed under this category of bio-nanocarriers. To serve the purpose of tumor-targeting efficiently, such bio-nanocarriers may be either of the following sub-types: conjugate (drug-biopolymer), nano-reservoir (biomolecule used as a corona/surface coating), or nanomatrix (nanosystem is entirely fabricated from the biomolecule). In many of the cases, the bio-molecules themselves possess some anticancer properties. Considering the limitations of the review, only some of the studies employing novel biological ligands have been prioritized and briefed upon. Some novel biomolecules that are intensively researched upon for serving the purpose of active targeting through the bionanotechnology approach include the antibody/affibody [116, 117], lipoproteins [118], proteins and peptides [119–121], pulmonary surfactant, and biosurfactant [122–126], vitamins and other small molecules [127]. The basic nanocarriers that can be modified using any of the aforementioned biomolecules for diverse oncological applications can be summarized as lipid-based nanoparticles (liposomes, SLNs, NLCs) [128–130], polymeric nanoparticles (dendrimers [131–134], polymeric [135–140], polymersomes [141–143], layer-by-layer nanoassembly (LBL) [144, 145], nanosponges [146–148], lipid-polymeric nanoparticles [149, 150], protein nanoparticles (casein, zein, etc.) [151], polysaccharide nanoparticles (mannosylated, chitosan, hyaluronan, fucoidan-based nanoparticles) [152, 153], carbon nanostructures (nanotubes, graphene, fullerene, nanodiamonds [154], inorganic nanoparticles (SPIONs, upconversion nanoparticles, silver nanoparticles, gold nanoparticles, mesoporous silica nanoparticles), and quantum dots [155–159]. Some other less explored nanostructured materials such as the biomolecular self-assemblies and nanofilaments have also been reported for the management of malignancies such as cancer [135, 160–164].

#### Antibody, Antibody Fragments, and Affibody

Antibodies or immunoglobulins (Ig) are specialized glycoproteins expressed over the surface of B cells to act as antigen receptors [165]. Amongst five major types of Ig (IgG, IgE, IgM, IgA, and IgD), IgG is most abundantly found in the serum and has been extensively researched for immunotherapy, vaccine production, and nanoparticle functionalization. While bioengineered Ig has been employed for immunotherapy of cancer since long, their use as targeting agents has emerged lately [166, 167]. Antibody-functionalized nanoparticles have been reported for targeted therapy of various types of cancers including lung cancer [168–172]. However, despite improvement in pharmacokinetics and biodistribution, diffusion of full-length antibody largely limits the intratumoral penetration of the payload. Several monoclonal antibodies (MAbs) have been bioengineered for immunotherapy of cancer including lung cancer.

To combine the advantages of tumor-specificity and higher intratumoral penetration through improved tumoral diffusion and cellular uptake, small fragments of antibodies are now gaining greater attention [173, 174]. The use of Ab fragments also overcome the Fc-induced bystander activation, as observed in the case of the conventionally used antibodies [116]. The most commonly used Ab fragments that are employed for drug targeting are a single-chain viable fragment (scFv) and antigen-binding fragment (Fab), respectively. The Fab fragments are generated through enzymatic proteolysis at the hinge of the whole Ab, resulting in its fragmentation. However, the scFv fragment, consisting of peptide-linked VL and VH, is produced employing Ab engineering techniques like ribosome display or phage display [175]. However, the Ab fragments lack immunogenicity and have low circulation half-lives due to their low molecular weight and lack of the Fc component. Strategies such as PEGylation and recombinant fragment fusion may be employed to overcome these limitations [176, 177]. Nanoparticles can also be conjugated with Ab or Ab fragments through functionalization methods that employ adapter molecules, covalent binding, or conjugate through adsorption to form bio-nanocarriers, thereby enhancing the targeting ability while minimizing their immunogenicity. Some of the factors to be considered while the formation of these bio-nanocarriers are: density of Ab per nanoparticle, steric orientation, and bond stability to ensure good in vivo performance and bioavailability [156]. Many research works utilizing antibody fragments for oncological applications have been reported in the literature [172, 178, 179].

Yet another bioapproach of a similar kind is the use of affibodies for targeted therapy of cancers. Affibodies represent a distinct class of bioengineered affinity proteins of low molecular weights (6.5 kDa) with diverse diagnostic, therapeutic and biotechnological applications [117]. Affibodies have high specificity and affinity toward definite protein targets and can be engineered to target different types of proteins. Affibodies are actually, mutated derivatives of the B-domain of the Ig-binding region of staphylococcal protein A (also referred to as Z-domain) [180]. This bioengineered Z domain displayed a high affinity for the Fc part of the Ab, but the affinity toward the Fab region was almost entirely deactivated. Affibodies targeted toward proteins like fibrinogen, insulin, transferrin, tumor necrosis factor-a, CD28, gp120, IL-8, EGFR, IgM, IgE, IgA, serum albumin, HER2, etc., have been studied extensively [117]. Affibodies have been extensively employed for imaging and optical imaging purposes, for affinity chromatography-based diagnostics, and for targeting payloads through bio-nanocarriers to tumors. Bio-nanocarriers employing the virus-derived affibodies, liposome-conjugated with affibodies “affisomes”, etc., for oncological applications have been discussed elsewhere in detail [117, 181]. Some examples relevant to lung cancer management have been discussed in the forthcoming section.

#### Lipoproteins

Lipoproteins are a group of endogenous nanoparticles composed of phospholipids, amphiphilic apoproteins, and neutral lipids. The use of lipoproteins as a carrier for diverse payloads can be attributed to its role as an endogenous bio-carrier for the delivery of hydrophobic cargoes like triglyceride and cholesterol. Their specialized bio-synthetic, bio-transport, and biodegradation pathway make them an attractive carrier system for specialized applications. Oncotargeting via lipoproteins is an attractive strategy because of (1) increased demand of lipids for cancer cell membrane synthesis causing overexpression of their receptors, (2) pharmacological substantiation of vascular drug-lipoprotein interaction, (3) epidemiologic association of cancer and lipoproteins, (4) bi-directional signaling and signal transduction mediated by lipoproteins and lipid rafts. This was evidenced by the involvement of the lipid rafts in H-RAS, Insulin-like growth factor (IGFR), EGFR, and the Hedgehog-mediated signaling pathways, which are majorly involved in malignancies [182]. However, it has been reported that only the HDL and LDL are suitable for oncological applications and delivery through bio-nanocarriers due to their unique alpha-helical protein-intercalation. This is because the other lipoproteins have larger lipid curvature and large diameter, which exposes them to water and renders them unstable [118]. Novel types of bio-nanocarriers that employ lipoprotein as targeting ligand through conjugation are being explored more recently for the treatment of cancer. The modification methods that are employed for the fabrication of such bio-nanocarriers include covalent alteration of proteins or the phospholipids, non-covalent surface loading, and reconstitution-mediated core loading [118, 182–185]. The application of such bio-nanocarriers in the context of lung cancer management has been briefed upon in the upcoming section.

#### Protein/Peptides

Protein and peptide bio-nanocarriers are one of the most common forms of bio-hybrids. Being one of the most ubiquitous biomolecules, the proteins and peptides have multimodal roles in the management of various diseases including lung cancer. Besides being employed as biomarkers [186–189], the proteins and peptides also have therapeutic [190–194] and drug delivery applications in lung cancer [119, 120, 151, 195, 196]. Due to significantly greater biocompatibility and biodegradability, as compared to the synthetic nanoparticles, protein-based nanocarriers, composed of albumin, gelatin, gliadin, elastin, zein, casein, β-lactoglobulin, soy protein, and whey proteins, have been employed since long for drug delivery applications in oncology [151]. Besides being employed as a nanocarrier and nanocages, the glycoproteins like transferrin- and apoferritin-based carriers are also employed as targeting vector and theranostic agents/nanoprobes [157, 197–199]. However, due to high molecular weights, poor in vivo bioavailability, and difficulty in conjugation to nanoparticles, peptides are being preferred over proteins for drug targeting. Recently used peptides for lung cancer targeting include the cell-penetrating peptides, iRGD peptide sequence, bombesin peptide, somatostatin peptide sequence, FSH-33 (Follicle-stimulating hormone analog), fibroblast growth factor peptide (tbFGF), peptides targeting epidermal growth factor receptor (EGFR), peptide GFE, peptide F3 [200]. A novel 7-mer peptide termed as “I-peptide” that mimicked carbohydrates and inhibits carbohydrate–mediated cell localization was devised by Hatakeyama et al. for the treatment of various types of cancers including lung cancer [201]. Bio-nanocarriers employing nanoparticles modified with such targeting moieties for lung cancer management have been elaborated in the forthcoming section.

#### Carbohydrates and Polysaccharides

Various carbohydrate- and polysaccharide-based nanodelivery systems have recently emerged as an important platform for oncotargeting. Due to high biostability, safety, biocompatibility, and low toxicity, it has gained considerable interest amongst scientists. Just as in the case of the protein-based delivery platforms, this platform is being shared for the fabrication of nanoparticles as well as for targeting purposes. Nanoparticles composed of or modified by chitosan, fucoidan, glycosaminoglycan, cellulose, galactomannans, pectins, starch, dextran, alginate have been reported for cancer targeting [153, 202, 203]. Several novel polysaccharide-based nanosystems are recently being developed for devising active targeting to the solid tumors. The lungs being macrophage-rich organs have numerous mannose receptors (MRs) and GLUT1 receptors on their cell surface, which favor the translocation and endocytosis of polysaccharide-modified/carbohydrate-modified nanoparticles into the tumor tissues [202].

#### Aptamers and Aptasensors

Bio-functional class of oligonucleotides with distinctive 3D conformations that have been engineered using a combination of in vitro selection and systematic ligand evolution methods to confer high specificity and affinity toward specific targets are termed as ‘aptamers’ [204]. With gaining pace in bionics and bioengineering, the aptamers are being explored for their oncological applications. The process of bioengineering and aptamer designing is largely facilitated by an in vitro selection method known as the ‘systematic evolution of ligands by exponential enrichment’ (SELEX). Due to significant molecular recognition capability, aptamers are being employed for functionalizing nanomaterials, nanoparticles, and fabricating devices like aptasensors. Aptasensors are biosensors fabricated using specific aptamers. Based upon mechanistic recognition, aptasensors are of the following subtypes: (a) sandwich-type, (b) split-type assay-based, aptasensors based on (c) cell membrane–aptamer interactions, (d) conformational switch, and (e) competitive binding of the aptamer to the target [205]. Several aptamer-based nanosystems that have been developed by researchers are discussed elsewhere in detail [206–213].

#### Pulmonary Surfactant and Biomimetic-surfactants

Pulmonary surfactant is an endogenous surfactant that is composed of phospholipids such as phosphatidylcholines and phosphatidylglycerols (90%) and a mixture of fatty acids, cholesterol, and surfactant proteins (10%) [214, 215]. The pulmonary surfactant is a complex lipoprotein, with unique interfacial properties that may serve to confer greater biostability and safety while predicting the inadvertent interactions of the pulmonary linings with the delivered nanoparticles. Some of the most commonly used pulmonary-derived surfactants, employed for formulating such bio-nanocarriers, are Alveofact®, Curosurf®, and Surfactin*®.* Bio-nanocarriers, composed of pulmonary surfactant, is an attractive strategy that has been attempted by several researchers for the delivery of various types of anticancer payloads to lung tumors [123–125, 216]. However, due to limitations associated with the mass production of the natural pulmonary surfactants, biomimetic pulmonary surfactants, and other biosurfactants have been developed and employed for similar purposes. The details of such bio-nanocarriers are referred to in the upcoming section [126, 217].

#### Vitamin and Other Small Molecules

Nano-biocarriers employing biomolecules like vitamins may be considered as the foundational-bioinspired nanocarriers. Led by the fact that receptors for some vitamins are overexpressed over the cancer cells, research interest grew in an entirely novel direction of active targeting to intracellular levels. Vitamins like folate, biotin, riboflavin, and tocopherol have been employed and extensively researched for their drug delivery applications due to their ease of conjugation over the nanoparticle surface, economic feasibility, their innocuous nature & high biocompatibility contributing a major share in the fabrication of the bio-nanocarriers [127, 155, 218–228]. Biotinylation is also an immensely useful tool for hybridization, bioengineering, and biomanufacturing of nanovesicles and bioderived cell/cell surface molecule or cell membranes. However, the success of vitamin-conjugated nanoparticles for active-targeted delivery is controversial due to their non-specific overexpression over many normal cells. This reason is likely to favor the use of other bio-molecules and bio-moieties over vitamins for more specific and targeted active-targeting. Other small molecules like anisamide and phenylboronic acid have also been extensively reported for designing cancer-targeted drug delivery in the literature [229–232]. But the constraints such as higher costs and biological instability of the small molecules greatly limit their further nanobiotechnological applicability.

### Novel Nano-biodevices

Nano-biodevices are the gadgets, equipment, contrivance, or their component (s) fabricated through multidisciplinary research in bionanotechnology for various clinical and biomedical applications [233]. Based upon the material of construction, there are four types of nano-bio-devices: microbial-based, immunosensors, DNA-based, and tissue-based nano-biodevices [234]. The biosensors, devised utilizing aptamers, have been discussed previously. Other nanodevices utilizing the function of membrane proteins have also been developed for diverse oncological applications [235]. With advances in bionanotechnology, several nanomaterials such as nanopillars, nanowalls, nanoballs, nanotubes, plasmon nanobubbles, and nanodots/quantum dots are being employed for the development of nano-biodevices such as nanowire arrays, nanobiosensors, nanochips, and nanobots, that have immense oncological applicability [233, 234, 236–240].

Considering the vastness of the topic and limitations of the article, only the nano-biocarriers and nano-biodevices that have been exploited for lung cancer management have been considered and discussed further.

## Bio-nanocarriers for Clinical Management of Lung Cancer

With several unaddressed oncological issues, the present oncological scenario demands an immediate pragmatist approach. Bionanotechnology emerged as a consequence of the convergent evolution of nanotechnology and biotechnology in oncological sciences to offer several advantages while overcoming their individual pitfalls. Bionanotechnology has assisted drug discovery and development in addition to quadripartite management of various types of malignancies including lung cancer.

### Bionanotechnology in Drug Discovery and Development

For understanding the complexity of cancer and underlying cellular and bio-molecular pathways involved therein, advanced nanobioengineered interventions are highly anticipated. Employing nanobioengineering-based tools have aided the process of drug discovery and drug development at several stages. Identification of molecular and genetic abberations in lung cancer has led to the development of several targeted therapeutics, also termed as “precision or targeted therapy”, which is highly specific for cancer cells. For example, the identification of EGFR led to the development of tyrosine kinase inhibitors, cetuximab (KRAS-interfering monoclonal antibody), VEGFR-targeted monoclonal antibodies such as bevacizumab and ramucirumab [6]. Several nanobioengineering-based tools like nanoproteomics, nanogenomics, nanosensors, nanoprobes, and cantilevers have contributed immensely to tracing biomolecular interactions and facilitated the identification and validation of novel targets. Discovery and identification of novel cellular, bio-molecular and molecular targets may help overcome the limitations of existent therapeutics like resistance and non-selective cytotoxicity. The next step of lead identification is facilitated with the use of bio-nanotools like nanobiosensors, nanoscale endocytosis assays, nanowire devices, nanoflow liquid chromatography, nanofluidics, nanobiochips, nanoarrays, and surface plasmon resonance. Furthermore, the process of lead optimization is facilitated with the use of quantum dots, inorganic nanoparticles, bio-nanocarriers employing small biomolecules attached to the nanoparticles, and fluorescence planar waveguide technique [241].

### Bio-nanocarriers for Secondary Prophylaxis of Lung Cancer

Secondary prevention/prophylaxis refers to the application of clinical and pharmaceutical interventions for prevention, suppression, or reversal of oncogenic progression from a premalignant condition to invasive malignancy [242]. Secondary prevention mode is meant for switch maintenance, continuation, or post-front-line therapy for prevention of relapse and achievement of better prognosis. Despite several attempts, cancer chemoprevention prevails in an inconclusive state [242, 243]. However, nano-biocarriers have also been employed extensively in designing cancer vaccines and immunotherapeutics that have therapeutic as well as a prophylactic role in high-risk patients (malignant oncological conditions) [244].

Tecemotide (L-BLP25, StimuVax) is an ideal example of one such nano-biohybrid. Tecemotide is a lyophilized liposomal formulation (~ 150–180 nm diameter) composed of lipids (dimyristoyl phosphatidylglycerol, cholesterol, and dipalmitoylphosphatidylcholine), monophosphoryl lipid A, and BLP25 lipopeptide (mucin 1(MUC1 specific antigen)). The glycoprotein MUC1 was evidenced to be overexpressed and aberrantly glycosylated in the patients of non-small cell lung cancer. MUC1 is identified as one of the most prevalent cancer-associated antigens that are involved in the bio-signaling tumor cell survival, proliferation, and progression in many forms of cancers including lung cancer. This can be attributed to MUC1-mediated interplay with many cell surface receptors like tyrosine kinase. BLP25, being MUC1-targeted immunogen, and monophosphoryl lipid A, being a TLR4 agonist were observed to trigger Th1 polarization and CD8c T cell-mediated immune response. Clinical evidence of the safety, immunogenicity, and efficacy of Tecemotide in patients with stage III B locoregional NSCLC was found to be promising in terms of improved survival rates and for secondary prophylaxis in the clinical phase III trial. Tecemotide is currently undergoing phase III clinical trial for maintenance immunotherapy in the patients of colorectal cancer following surgical resection or hepatic metastases and as a maintenance therapy or adjuvant therapy post-chemoradiotherapy in the patients of stage III NSCLC [245–249].

Cancer vaccine development and cancer immunotherapy have gained a significant boost with an oncolytic virus-based bio-nanocarriers. ExtraCRAd is a unique multivaccination platform designed by Fusciello et al. for the prevention as well as treatment of melanoma and lung cancer. The ExtraCRAd nanoparticles are composed of bioengineered oncolytic virus Ad5-D24-CpG, encapsulated in cancer cell membranes. For the preparation of the ExtraCRAd vaccine, the polymeric membrane extrusion method was employed. This strategy synergizes the immunogenicity of the tumor-associated proteins present in the cancer cell membrane with oncolytic and adjuvant properties of the oncolytic viruses. The ExtraCRAd displayed a significant increase in the infectivity and was observed to elicit CD4^+^ and CD8^+^ mediated by an antitumor response in a solid tumor model. Moreover, ExtraCRAd may be personalized for immunotherapy of different patients through the biopsy-derived tumor cell membrane. Excellent tumor regression was observed in vivo, both, in preventive and therapeutic preclinical settings. The scientists foresee such nano-biocarrier to be a promising platform for personalized vaccination and prevention [250].

Yet another nano-biohybrid platform that facilitates intranasal administration of the recombinant CCL21 gene for prophylactic immunotherapy has been proposed by Kar et al. The researchers have developed EGF-appended CCL21 vault nanocapsules from Chalmydial membrane protein that can target the tumor cells overexpressing EGFR receptor and elicit an immune response through the phosphorylation of Tyr1173. The CCL21 nanovaults were observed to partake in chemotaxis and promote leukocyte infiltrates such as DEC205+ DC, CCR7^+^T, CXCR3^+^T, and IFNc^+^T lymphocytes. A significant tumor growth suppression was also observed with the CCL21 nanovaults in the preclinical trials. This result was justified by the observation of augmented splenic T cell activity that results in a systemic antitumor response, accompanied by a reduction in the immunosuppressive cells like the IL-10 T cells, regulatory T cells, and the myeloid-derived suppressor cells (MDSC). The preclinical results in a murine model demonstrated the efficacy of this approach for secondary prophylaxis and immunotherapy of several malignancies including lung cancer [245].

The clinical establishment of the anticancer activity of CpG-oligodeoxynucleotides (ODNs) in lung cancer has led to the development of several novel formulations for increasing its selective tumor targetability and cell uptake through the optimization of its pharmacokinetics and biodistribution. Several studies that utilize lipid-based delivery systems and virus-like particles have been employed by researchers to achieve the aforementioned goals while increasing their immunostimulatory action [251–253]. A review of the lipid-based bio-nanocarriers of CpG ODNs (LCpGODNs) has presented the immense potential associated with their use as an immunostimulant anticancer immunotherapeutic and vaccine. The review also reports studies that have observed strong elicitation of immune response mediated by CD8^+^ T cells after the administration of LCpGODNs, suggesting the role of lipids as an adjuvant. The strategy was reported to defend against the xenogeneic antigens while sensitizing the immune cells to attack the self-antigens like glycoprotein 100 and TRP2 in a pulmonary metastatic B16 model. This presents an immense potential to target aggressive tumors expressing even very low MHC class I levels [252].

**Fig. 3:**
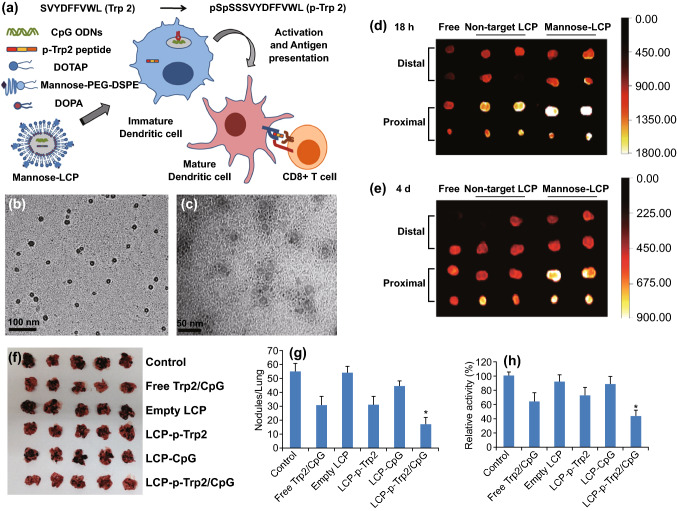
**a** Mannosylated LCP-nanoparticle (MN-LCP-NP)-based vaccine for dendritic cell (DCC) activation. DC activation results in inducing cytotoxic T-cell-mediated immune response. TEM microphotographs of **b** mannosylated nanoparticles and **c** p-Trp2 peptide and CpG ODN-loaded MN-LCP-NP. Scaling = 100 nm and 50 nm respectively. **d** Biodistribution in lymph nodes of the vaccine formulation upon s.c administration in C57BL/6 mice containing ^125^I-labeled p-Trp2 peptide and Texas Red-labeled ODN. Free: in 5% glucose; Non-target LCP-NPs, MN-LCP-NP, LCP NPs without DSPE-PEG-Mannose; and with DSPE-PEG-Mannose. Readings noted after 18 h and **e** after 4 days. **f** Lungs isolated from C57BL/6 mice with B16F10-luc cells induced lung metastatic melanoma after being treated with different formulations. **g** Tumor nodules **h** luciferase activity in lungs (n = 5, *P < 0.05). Adapted from with permission from Ref. [254] Elsevier, Copyright 2013

A similar research work that reports the use of mannosylated lipid-calcium-phosphate nanoparticles (LCP) for facilitating intracellular co-delivery of CpG ODN and Trp2 peptide (Trp2-P) to lung cancer cells has been reported by Xu et al. This novel nano-biohybrid was observed to augment the Trp2-P-specific immune response, mediated by MHC-I restricted T lymphocyte and IFN- γ in B16F10 lung metastatic model. Such mannosylated nanoparticle-based vaccines may facilitate dendritic cell delivery and can stimulate immune response directed to cancer cells (Fig. 3) [254].

Research by Chen et al. has demonstrated the use of bacterial (Salmonella) outer membrane vesicle (BOMVs) for secondary prophylaxis to metastasis and chemo-immunotherapy of lung cancer. This novel nano-biohybrid consists of tegafur-loaded polymeric micelle core, coated over with BOMVs. The BOMVs were designed to induce an immune response by sensitizing the cytotoxic T lymphocytes–mediated immune response to target the tumor cells directly. The antitumor efficacy and antimetastatic activity of BOMVs were apparent from the results of the preclinical in vivo data in a murine model of melanoma. A remarkable increase in the survival rates, tumor inhibition, and antimetastatic potential were observed with the BOMVs, suggesting their use as a versatile nano-biocarrier [255].

An entirely novel bioinspired approach was adapted by Chen et al. for the prevention of pulmonary metastasis of melanoma. The researchers developed a TRAIL-decorated nanovector composed of high-density lipoprotein (HDL) for targeting mesenchymal stem cells (MSCs) that overexpress scavenger receptor B type I (SR-BI). The pDNA that encodes the genetic expression of tumor necrosis factor (TNF)-related apoptosis-inducing ligand (TRAIL) was used as a vector for targeting the pulmonary MSCs. For the construction of this bioinspired nanovector, pDNA was electrostatically conjugated to cationic copolymer, composed of polyethyleneimine and lauric acid. The developed nanoparticles had uniform particle size and high in vitro stability in plasma. The nanovectors had high transfection ability and cell uptake by the MSCs targeting B16F10 (melanoma) cells through a highly targeted therapeutic approach. Such an approach not only prevents side effects but also prevents metastasis of the melanoma cells [256]. Another vaccination approach employing tumor cell lysate-encapsulated bacterial nanoghosts was reported for preventive immunotherapy against many forms of cancers including lung cancer [36, 257].

The research works discussed in this section are only a few of the researches that exemplify the emerging prophylactic role of bionanotechnology in pulmonary oncology.

### Nanobiodiagnostics in Lung Cancer

Diagnostics in cancer involve two different aspects: (1) bio-imaging and (2) identification of tumor-associated molecular markers. Conventional diagnosis of lung cancer is done employing diagnostic methods such as sputum cytology, imaging tests like CT scan, thoracic X-ray, PET and biopsy techniques such as bronchoscopy, thoracentesis, endoscopic ultrasound and open biopsy. The prevalent cancer diagnostic methods have limitations like poor image resolution, lack of specificity, low detection sensitivity, low signaling strength, and limited tumor-associated molecular marker identification techniques. In some cases, imaging methods have also been associated with toxicity issues [3]. Several genetic, epigenetic, proteomic, metabolic bio-molecules that are increasingly being recognized as biomarkers comprise mutated DNA/matrix RNA expression, altered/methylated DNA, altered protein levels, qualitative and quantitative alterations in low molecular mass metabolites [258–263]. Exosomal miRNAs and miRNAs, circulating tumor cells (CTCs), tumor stromal cells, endothelial cells, and immune cells are also gaining increased attention to be used as a biomarker for the detection of tumors. Tumor-associated bio-molecules like methylated sputum microRNAs/DNA, metabolome, microbiome, and airway epithelial cell biomarkers can be detected through several diagnostic methods upon screening of the respiratory tract. Peripheral tumor-associated bio-molecules like DNA methylation patterns of leucocytes, serum autoantibodies, microRNAs, CTCs proteomic signatures, and circulating cell-free DNA/circulating tumor DNA may also be screened upon for tumor detection [264–267]. These techniques provide a deeper understanding of the subtypes of lung cancer and aid in deciding therapeutic interventions thereof, wherefrom the concept of personalized therapeutics has emerged. This has even led to the development of tyrosine kinase inhibitors for targeting the patient populations with molecular aberrations in the EGFR and ALK genes. Novel bio-diagnostic methods like chromogenic/fluorescence in situ hybridization or PCR-based multiplexed techniques like SNap-Shot or mass spectroscopy-based genotyping-based methods like the Sequenom MassARRAY, Sanger sequencing, and restriction length fragment polymorphism have considerably revolutionized the diagnostic field [268–270]. Comprehension of sub-variants of lung cancer at genetic/molecular levels can help in devising novel personalized therapies. However, due to a large number of molecular aberrations involved in lung cancer, high-throughput screening methods capable of exome/genome/transcriptome sequencing are being developed [271]*.* The application of nano-bio-based detection and imaging techniques may facilitate high-throughput screening of biological samples, novel biomarker identification, biomarker detection with high specificity at low detection levels, and molecular mapping [114, 186, 187, 240, 265, 272–274]. Several attempts are being made for developing multifunctional molecular nanoprobes that may clarify the functionalization of biological systems at the nanometer scale, identify the process of their integration within the cells allowing recognition, thereby prompting spatiotemporal signals that are involved in cell–cell and cell–space interactions. Futuristic nano-biodevices attempt to develop tools that may dynamically track and observe cellular and biomolecular interactions, biodistribution, bio-kinetics, and bio-interaction of various therapeutic and diagnostic agents. Other unmet objectives such as bioengineering of encodable genetic markers and single-molecule analysis and bio-fate analysis are also being worked upon by the bionanotechnology researchers [4]. Quantitative analytical techniques and information integration techniques with bioengineered nano-tools may be of great resort to the researchers in the development of multifunctional devices that not only sense the external and internal bio-signals but also regulate cellular responses like proliferation, differentiation, apoptosis, etc. Information integration from biotechnologically advanced “omics” techniques may be computed using predictive tools and may be employed as virtual models for extrapolating the spatio-mechanical, multimodal, and ordered assemblage of biomolecules and synchrony of several bioprocesses [4]. Some examples of bio-nanocarriers that have been researched upon with similar objectives have been discussed hereafter.

While metal-based nanoparticles have significantly contributed to the enhancement of conventional CT scanning techniques, most of them do not facilitate targeted imaging. To facilitate the targeted imaging of lung cancer, a novel nano-bio-hybrid platform was developed by Xu et al. The authors of this research have discussed the development of NJ0001-coated nanomagnetic beads targeted to the SP70 antigen that is overexpressed in the lung adenocarcinoma cells. NJ001 is a monoclonal antibody, directed to the SP70 antigen. Strong signaling intensities were observed in an orthotopic mice model upon a micro-CT scan. The NJ001 can also be labeled with a NIR-florescent dye CF750 to serve as a probe for molecular imaging using an anti-SP70 fluorescent imaging technique. With SP70-targeted bio-nanocarriers, it was possible to detect pulmonary lesions two weeks earlier than the detection in control. Such preclinical findings indicate the NJ0001-labelled probe to be an excellent tool for facilitating early detection of lung cancer through molecular imaging [275].

Due to the short biological half-life of the conventionally used PET tracers and their constrained diagnostic ability in various types of lung cancer, novel nano-approaches are being explored [3]. Cai et al. have reported the development of a nano-bio-hybrid system to facilitate imaging of tumors with a high spatial resolution with combined PET and CT imaging techniques. Such an imaging platform can combine the high spatial resolution of CT imaging with high sensitivity of the PET imaging techniques. To facilitate precise simultaneous PET/CT imaging, self-assembled lipidic nanocapsules encapsulated with iodixanol were tagged with self-chelated ^64^Cu and targeted to the tumor cells using the GLT21T aptamer. GLT21T is an aptamer targeted to tyrosine kinase receptor AXL that is overexpressed in lung cancer. GLT21T-conjugated lipid nanocapsules were formulated using self-assembling of the Chlorin e 6 (Ce6)-conjugated lysophosphatidylcholine (LPC). The ^64^Cu moiety was contained at the center of the Ce6 tetrapyrrole ring, by its self-chelating ability followed by loading of iodixanole into its cavity. The iodixanol loading can be modified to meet the imaging requirements. The bio-nanocarriers thus formed were assessed in the A549-orthotopic mice model and compared to the conventional PET and CT imaging results. It was reported that the microscopic tumors (~ 500 μm) that were undetectable using the conventional PET and CT imaging techniques were detectable at high resolution, contrast, and sensitivity by employing such a dual-mode imaging nano-bio-hybrid tool [276].

**Fig. 4:**
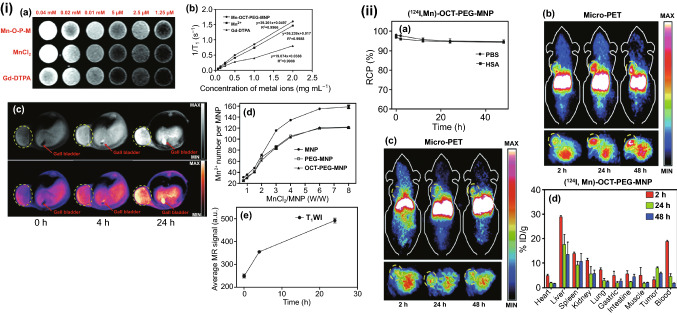
**(i)** In vitro* and *in vivo MRI analysis of Mn^2+^—OCT-PEG-MNPs: **a** T1-weighted MRI images at 1.5 T, TR: 531 ms, TE: 9.1 ms of Mn^2+^ -OCT-PEG-MNPs (upper panel), MnCl2 (middle panel), Gd-DTPA (bottom panel) with various concentrations (2.0, 1.0, 0.5, 0.25, 0.125, and 0.0625 mg mL^−1^. **b** in vitro T1-linear relaxation rates (1/T1, s^−1^) of Mn-OCT-PEG-MNPs, Mn^2+^ and Gd-DTPA measured. **c** Axially imaged T1-weighted MR images of NCI-H469 mice ((tumor site enveloped by a yellow dotted line, and gall bladder was indicated by a red line) before and after i.v, tail injection of Mn^2+^-OCT-PEG-MNPs(TR: 531 ms, TE: 9.1 ms. **d** Relationship of Mn^2+^ attached on one MNP, PEG-MNP, and OCT-PEG-MNP and feed ratio (WMn/WMNPS). **e** Quantitative analysis of signaling intensity at the tumor site at various time intervals. **(ii)** 5 PET analysis of ^124^I labeled Mn^+2^-OCT-PEG-MNPs. **a** In vitro stability analysis of (^124^I, Mn^+2^)-OCT-PEG-MNPs in 5% HSA solution and PBS (pH = 7.4). **b** The sagittal plane (upper panel) and transaxial (bottom panel). Micro-PET images of NCI-H69 tumor (tumor site enveloped by a yellow dotted line) scanned at 2, 24, and 48 h after tail vein injection of (124I, Mn^+2^)-OCT-PEG-MNP. **c** Micro-PET imaging of A549 tumor mice at 2, 24, and 48 h after tail intravenous injection of (124I, Mn^+2^)-OCT-PEG-MNPs. **d** Biodistribution of (124I, Mn^+2^)-OCT-PEG-MNPs in NCI-H69 tumor mice (n = 3) measured by gamma scintillator at 2, 24, and 48 h after tail vein injection (data expressed as % ID g^−1^ ± SEM. Adapted from Ref. [278] with permission from The Royal Society of Chemistry, Copyright 2019

Magnetic resonance imaging (MRI) is not a favored diagnostic method for lung tumors due to biological factors like low intrapleural proton density, high mediastinal and thoracic movements, and heterogenic pleural magnetic distribution [277]. A novel nano-biohybrid was developed by Xia et al. to facilitate the multimodal (MRI imaging and micro PET) imaging of lung cancer. Bio-nanocarriers composed of octreotide-modified organic melanin nanoparticles (OC-MNPs) targeted to somatostatin receptors (SSTR2) were developed for photoacoustic imaging of pulmonary tumors. SSTR2 are highly overexpressed in many forms of lung cancers that are difficult to be diagnosed at early stages. The magnetic resonance contrast agents like Mn^+2^, ^124^I, and N-Bromo succinimide can be chelated with MNPs directly to facilitate photoacoustic imaging (PI) also. The nanoprobe displayed assertive results when evaluated for imaging ability through PET, MRI, and PI in the xenograft model of NCI-H69 tumor overexpressing the SSTR2 receptors (Fig. 4). A remarkably higher T1-weighted signaling intensity was observed in the tumors on the PI imaging of tumors after 24 h on the administration of Mn^+2^- modified OC-MNPs, as compared to the prescan control groups. The ^124^I, Mn^+2^-modified OC-MNPs were observed to have significantly higher cell uptake in the NCI-H69 tumors as compared to the SSTR2-negative A549 cells (8.03 ± 0.37% ID g^−1^ as compared to 3.35 ± 0.54% ID g^−1^). With favorable preclinical results, the OC-MNPs nanoprobe may be considered further for clinical trials [278].

Chen et al. developed a novel nano-biohybrid-based multivalent probe for the detection of metastatic lung cancer cells. Click chemistry-based reaction was employed to synthesize galactoside-capped gallamide with triantennary dendritic morphology, containing a thiolate focal group. While the core of the nano-biohybrid was composed of fluorescent CdSe/ZnS, the dendron formed its hydrophilic shell. The nano-biohybrid was evidenced to undergo high cell uptake via asialoprotein receptors that are overexpressed on lung cancer cells. The applicability of such a nano-biohybrid in photodynamic therapy has been evidenced by their uptake in an active mitotic state as well as non-mitotic lung cancer cells. The developed multivalent probe was reported to be a useful tool for studying the process of cell adhesion, cell identification, endocytic mechanisms, and multimeric carbohydrate interactions [279].

Despite having maximal permissible exposure and deeper laser penetration, most of the NIR-II-active nanomaterials face constraints about their tumor-targeting ability and imaging ability. A novel nano-biohybrid, comprising of macrophages loaded with bimetallic (FePd) nanoparticles, was developed by Yang et al. to facilitate targeted phototherapy in NIR-II biowindows and MRI imaging of various types of tumors. The developed nano-biohybrid was evaluated for their in vitro and in vivo photothermal activity and MRI imaging ability, wherein they showed excellent tumor targeting, good imaging ability, and photothermal conversion efficiency of 36.7%. A tumor volume reduction of ~ 90%, with insignificant organ toxicity, was observed with the use of this nano-biohybrid as compared to the control group. The observed results present an excellent platform that can be explored further clinically for combining photodynamic therapy and MRI imaging [280].

With the advancing era of microfluidic-based nanomaterials, several nano-biodevices have emerged. Examples of such devices have been reported for the detection and diagnosis of serum biomarkers of lung cancer [281]. Gao et al. have devised CMOS-compatible silicon nanowire (SiN)-based multiplexed detection nanoarray for multiplexed detection of lung cancer biomarkers. The SiN nanosensors can be combined with a microfluidic device to enhance the performance of the nanodevice through rapid analysis of small sample volumes and allow multiplexed detection of the biomarkers of lung cancer such as miRNA-126 and CEA. Besides offering a sensitive real-time detection ability and offering single-molecule detection ability, these SiN nanosensors can be produced in bulk using the CMOS-compatible anisotropic self-stop etching method, thus providing an immensely useful method for clinical diagnosis of cancer as well as other diseases [240].

Some other examples of bio-nanotools that have been employed for diagnostics of lung cancer are summarized in Table 3.

**Table 3:** Bio-nanotools for lung cancer management

Onco-modality	Bio-nanotools	Key findings	References
Diagnostics	Nanocomposites with quantum dots and biotinylated *Magnetospirillum magneticum*-derived magnetic particles	Fluorescent and magnetic tagging of primary cancer cells and the circulating tumor cells were nano-biohybrid facilitated. Due to multiple surface expression of enzymes, proteins like protein G over the bacterial cell wall, the method can be employed for designing various cancer bioassays and imaging	[34]
	Viral particles encapsulated PEG-self-assembled CdSe/ZnS quantum dots (QDs)	The nano-biohybrid, composed of bromoviridae-derived viral capsid encapsulated QDs, provided high biostability with high intracellular cell uptake with minimal leaching of photoreactive products upon irradiation	[56]
	Nanodevice based on nanoporous glass (NG)-integrated volumetric bar-chart chip (V-Chip)	The ELISA-based detection device (NG-V chip) can be surface-modified for the detection of lung cancer biomarkers such as SCCA, CEA, and CYFRA 21–1 with high sensitivity. Rapid diagnosis of the device facilitates economical personalized cum point of care diagnosis in cancer patients and may be used for multiple cancer biomarker detections	[281]
	Gadolinium (Gd_2_O_3_)-doped carbon-11-choline‑Lenvatinib (GdCo @ Ln) nanoparticles	GdCo @ Ln-nanoparticles had clinically significant contrast for imaging and diagnosis, which facilitated PET imaging of lung cancer and significantly improved the survival in patients, thereby helping devise novel strategies for targeting of the malignancy	[70]
	Bacteriophage T4-fluorescent dye nanoprobe	The nanobiohybrids composed of tail-less bacteriophage T4-labelled with Alexa Fluor 546 and Cy3 fluorescent dyes were observed to give fluorescent signal enhancement of ~ 90% as compared to free counterparts. The nano-biohybrid had high intracellular stability and may be applied as a molecular nanoprobe for flow cytometry and cell imaging	[329]
*Secondary prophylaxis/metastasis prevention*	Hepatitis B core Ag or the bacteriophage Q*β*-derived virus-like particles-encapsulated CpG motifs ( CpG-VLPs)	A potent CD8^+^T cell-mediated immune response against vaccinia virus and solid fibrosarcoma cells was observed on in vivo assessment of the vaccine composed of the CpG-VLPs vaccine	[251]
	PEGylated liposome-polycation-DNA complex (P-LD) for si-RNA-mediated tumor targeting	The nano-bio-hybrid improved the tumor localization and siRNA-mediated gene silencing (threefold higher) through selective survivin downregulation in lung cancer cells. P-LD-treatment in H460 cells was reported to have potent antitumor activity as reflected by high translational ability and 90% apoptosis, which was fourfold higher than the unencapsulated, non-targeted siRNA-treated H460 cells	[330]
*Mode of treatment*			
(a) Chemotherapy	Lung biosurfactant mimetic inhalational particles encapsulating paclitaxel	Pulmonary surfactant biomimetic surfactant composed of phosphatidylcholine (DPPC) and dipalmitoyl phosphatidylglycerol (DPPG) was used to deliver paclitaxel (PTX) after conversion to an inhalational dry powder. In vitro cell line analysis of the formulation demonstrated high cytotoxicity upon cell viability assay, fluorescent microscopy and at air-interface transepithelial electrical resistance analysis	[331]
	Doxorubicin nanoparticles, surface conjugated with GE-11 peptide	GE 11 peptide targets the EGFR cell receptor over lung cancer cells with high specificity. In vitro cell line assays on A549 cells revealed that the liposomes with 10% of GE-11 coating demonstrated high antitumoral activity as apparent from the 2.6 times lowered IC_50_ values when compared to the nontargeted PEGylated liposomes. EGFR-mediated cell uptake (mediated through clathrin-endocytosis) was significant from flow cytometry and fluorescent microscopy	[332]
	Daunorubicin (DN)-encapsulated apoferritin nanocages conjugated with hyaluronic acid (HAA)	Cationic cytotoxic agent DN was loaded by combining it with negatively charged poly-L-aspartic acid (PLA) through electrostatic interaction in apoferritin nanocages, surface conjugated with HAA to target the CD44 receptors, overexpressed on lung cancer cells. The in vitro and in vivo analysis demonstrated high biostability and low systemic toxicity of the nano-biohybrid	[49]
	Anti-carbonic anhydrase IX (anti-C IX) antibody and cell-penetrating peptide (CPP33) dual-ligand modified triptolide-loaded liposomes (A-CPP-TL-LP)	The A-CPP-TL-LP demonstrated high in vitro cytotoxicity and good tumor penetrability in the C IX-expressing 3D tumor spheroids. In vivo pharmacodynamic studies in orthotopic mice model of lung tumor demonstrated no systemic toxicity and significant cytotoxicity after pulmonary administration	[333]
(b) Phototherapy	Porphyrin-high-density lipoprotein (HDL)nanoparticles	Scavenger receptor class B type I (SR-BI)-targeted porphyrin HDL-mediated phototherapy is a novel approach toward photothermal ablation of tumors. Upon irradiation at 671 nm laser, the porphyrin HDL nanoparticles were observed to have high therapeutic efficacy when evaluated in H 460 cells. The in vivo assessment in the lung tumor model showed 73% cell apoptosis with no signs of toxicity to normal adjacent tissues	[334]
	Erythrocyte cell membrane-inspired bovine serum albumin (BSA) co-encapsulated with 1,2 diaminocyclohexane-platinum (II) (DACHPt) and indocyanine green (ICG), i.e., (BS@ Pt-ICG-NPs)	The BS@ Pt-ICG-NPs released payload upon NIR irradiation and exhibited high anticancer activity mediated by singlet oxygen, reactive oxygen species (ROS) and photosensitization to have simultaneous anticancer activity	[335]
	Platelet-membrane (PLM) cloaked hollow nanoparticles of bismuth selenide (HNBS) for delivery of ICG	PLM conferred high tumor homing ability, prolonged systemic circulation and precluded non-targeted drug release. The HNBS had high ICG loading ability and high stability under hyperthermia, proving an efficient means of tumor treatment	[336]
(c) Immunotherapy	T-cell labeled with gold nanoparticles and CT imaging for image-guided immunotherapy	Cancer cell-tracking and tumor-site accumulation of a high degree were achieved by employing these nanobiohybrids. Significant tumor regression was evident from proliferation assays and high amounts of cytokine release	[337]
	M2 peptide-functionalized -interference (RNAi)- nanoparticles (M2-RNA-NPs) for tumor-associated macrophages targeting	The M2-RNA-NPs- mediated dual-targeted immunotherapy to tumor-associated macrophages and cancer cells by gene silencing ability of the RNAi. Low dose administration of the nanohybrid showed synergistic anticancer activity with a significant reduction in the tumor size (~ 95%) and increased the survival rates by 75%	[338]
	MicroRNA-125b-encapsulated hyaluronic acid-PEI-nanoparticles targeted to TAMs (HA-miR-NP)	TAM targeting through HA-miR-NP mediate signal transduction for tumor proliferation, angiogenic and metastasis. Intraperitoneal administration of the HA-miR-NP demonstrated sixfold higher M1 to M2 macrophage ratio and about 300 times enhanced iNOS (M1 biomarker) to Arg-1(M2 biomarker) as compared to the control group	[339]
(d) Gene therapy	CDC20 siRNA-encapsulated cationic liposomes	Cell division cycle homologue 20 (CDC20) is a cell cycle regulator that is overexpressed in multiple forms of cancers including lung cancer. Encapsulation of synthetic CDC20 in guanidinylated amphiphilic cationic liposomes with stearyl chains was observed to inhibit tumor growth by cell cycle arrest at the G2/M phase and inhibit lung metastasis in C57BL/6 J metastatic lung cancer—mice model	[340]
	CD44-targeted–lipid-modified Hyaluronic acid-modified SSB/PLK1 siRNA-self-assembly nanosystems (HA@siRNA)	Hyaluronic acid was derivatized using lipids having polyamine functionalization and varying carbon chains and nitrogen contents to facilitate self-assembling and encapsulation of the siRNA. The cy3loaded nanosystem was observed to undergo high cellular endocytosis. The SSB/PLK1 siRNA encapsulated nanosystem showed CD44-specific gene knockdown in the primary lung cancer cells as well as the tumor-initiating stem cells	[341]
(e) Combination therapy	EGFR targeted—Bcl-2 siRNA and doxorubicin co-encapsulated mPEG-PLGA-PLL nanoparticles (E- BCL-DX-NPs)	E- BCL-DX-NPs were observed to undergo significant cell uptake and induce apoptosis in H1299 cells. In xenograft murine model, the formulation showed higher Bcl-2—mediated tumor suppression as compared to the unconjugated counterpart	[342]
	HA and tetraiodothyroacetic acid (TIA)—dual liganded solid lipid nanoparticles delivering DTX	HA serves to target the CD 44 receptors, while TIA targets the αvβ3 receptors that are overexpressed in lung cancer, while DTX has high anticancer activity. In vivo imaging and vessel, distribution tests showed high tumor-targeting ability. Significant tumor growth suppression and anti-metastatic effect were observed in the xenograft murine model	[343]
	Photothermally active bioinspired lipoprotein (BLp) nanoparticles for tumor priming to the secondary nanoparticle (BLp)	Administration of BLp to solid lung tumors disrupted the extracellular matrix (ECM) and tumor stromal cells (TSM), facilitating tumor priming to the secondary BLp nanoparticles. With this strategy, about 4.27-fold higher tumor penetration, 27 times higher tumor accessibility, and 97.4% higher anti-metastatic effects were observed on subsequent administration of the BLp	[344]
	Indocyanine green (IG) and imiquimod (IQ) co-loaded poly(lactic-co-glycolic) acid (PLGA) nano-biohybrid	Phototherapeutic agent (IG), Toll-like receptor agonist (IQ) in combination with the checkpoint-blockade by lymphocyte antigen-anti-cytotoxic T-4 (CTLA4) demonstrated higher checkpoint blockade and anticancer activity in mice tumor models	[345]
Theranostics	Folate-functionalized polyethyleneimine passivated-reducible carbon dots (F@P-CDs) loaded with siRNAs (EGFR and cyclin B1)	F@P-CDs showed a highly specific intracellular release of siRNA payload accompanied by blue photoluminescence upon irradiation at 360 nm in the acidic intracellular microenvironment. The in vitro cytotoxicity evaluation demonstrated high biocompatibility and highly targeted delivery ability of the siRNA- F@P-CDs nano-biohybrid. The multi-functionality of nanobiohybrids was apparent from the gene silencing ability, targeting ability, stimuli-responsive delivery, bioimaging ability, thus offering a promising theranostic tool for real-time monitoring and therapy of many forms of cancers including the lung cancer	[346]
	Octreotide-decorated honokiol and epirubicin-loaded liposomes (O-HN-Ep-LP)	Liposomes were surface decorated with octreotide for targeted drug delivery, while honokiol was encapsulated in the lipid bilayer to eradicate the vasculogenic mimicry channels and inhibit metastatic dissemination of the cancer cells and epirubicin was employed as a cytotoxic agent. The O-HN-Ep-LP were observed to have high in vitro cell cytotoxicity assays in Lewis lung carcinoma cells (LC). Molecular signaling involving MMP-2, PI3K, FAK, VE-Cadherin and apoptotic enzyme caspase 3 were modulated by O-HN-Ep-LP to exhibit high anticancer activity. The O-HN-Ep-LP demonstrated high in vivo safety and efficacy in the LC cell-induced mice model	[347]

### Nanobiotherapeutics in Lung Cancer

Nanobioengineering may not only assist targeted drug development as mentioned earlier but also facilitate targeted drug delivery. Idealistic drug delivery is characterized by features like prolonged systemic circulation, controlled release pattern, high biostability, targeting ability across various biological barriers, immuno-stealthing property in addition to protecting the payload. Such idealistic criteria have led to the development of targeted nano-drug carriers that assemble these virtues by the means of their modifiable dimensional properties, fabrication, PEGylation, and surface modification.

Nanotechnology has come up with several nanocarriers that have brought a paradigm shift in the field of oncotherapy. Targeted drug delivery in oncology is of utmost importance to overcome challenges associated with tumor targeting. Tumor-targeting may be achieved by the means of ‘passive targeting’ that operate through the EPR effect or by the means of ‘active targeting’ that operates via functionalization using targeting moiety/ligand. Given the complexity of tumor cytoarchitecture and barriers that prevent efficient antitumor activity, several shortcomings of the passive targeting strategies have been identified. These include a lack of selective cellular targeting and transitional hurdles across the tumor stroma. Such findings have led to the conceptualization of active targeting strategy as a futuristic mode of targeted drug delivery. The active targeting strategy can be employed for targeted delivery to extracellular tumor matrix, CTCs, lysosomes as well as mitochondria. Unlike passive drug targeting, active targeting operates via specific ligand–receptor interactions or biochemical mechanisms.

However, the real transformation of the active drug targeting approach is difficult due to challenges associated with oncotargeting. The factors such as proximity of the targeting moiety to the target, systemic clearance, unsolicited immune cell response, and immune cell uptake, nanoparticle characteristics, ligand density, non-specific biological/perivascular interactions, juxtaposing ligands at the target site, steric hindrance, unfavorable ligand orientation, etc., play a decisive role in active drug targeting of the nanoparticles [101].

Four generations of nanocarriers have evolved to date. The basic nanovescicular and nanoparticulate systems that aided solubility, conferred sustained/controlled release ability and bioprotection to the loaded cargo are considered as the first-generation nanocarriers. The second-generation nanocarriers are PEGylated nanocarriers that employ PEG as a ‘stealth’ molecule to prevent their immune recognition and RES uptake, thereby prolonging their systemic circulation and conferring passive targeting ability. Third-generation nanocarriers are complex nanocarriers that add to the virtues of the second-generation nanocarriers by offering ‘active targeting ability’, i.e., spatiotemporal delivery aided by the means of stimuli-sensitivity or through cell-targeted moieties. However, the in vivo performance of these active-targeted nanocarriers has been debated due to many factors [101]. The emerging concept of nanobioengineering has inspired researchers to design and develop novel fourth-generation nanocarriers, the bio-nanocarriers through bioengineering of cells and cell membranes or other biological components.

The majority of the active targeting strategies employed by researchers have been stratified as external stimuli-based, tumor microenvironment-based, and targeting ligand-based [282]. However, innovations in the nanobioengineering and nano-bioconjugation techniques have opened up several avenues in tumor targeting and have almost re-defined the terms of active drug targeting. Biocloaked, bioresponsive, bioinspired, biomimetic approaches of active drug targeting are recently gaining higher attention. The actively targeted nano-biocarriers have been reported to facilitate cancer cell targeting at the cellular/subcellular levels, to the tumor stroma, mitochondria, cancer stem cell, and tumor-associated cells [15, 74, 283–285]. Few other tumor-targeting approaches offered by bio-nanocarriers are enlisted below:

- Nano-biocarriers that combine stimuli-sensitive and tumor micro-environment-sensitive intelligent drug-delivery systems may be employed to facilitate spatiotemporal drug release along with the most accurate biochemical means of active targeting [286].
- Approaches like multistage targeting that are difficult for conventional drug delivery systems have been employed using the nanocarriers [287] and may be combined with biotechnology to help devise the nano-biocarriers.
- Overcoming multidrug resistance with the possible use of P-gp inhibitors and nano-biocarriers [122, 288–291].
- Nano-biocarriers may facilitate ratiometric and synergistic drug delivery [292] or dosage form designing in a metronomic mode [293].
- Nano-biocarriers also address recently discovered ‘on target but off the site’ like advanced drug delivery issues, faced by several target-specific drug categories (e.g., nano-biocarrier-based delivery of anti-PD-1 inhibitors [294].

Nanobioengineering and bionanotechnology have proved their versatility and multimodal applicability in drug delivery for multiple types of payloads like chemotherapeutic agents, phototherapeutics, genes, antibodies, small molecules, phytochemicals, microbubbles, imaging, and theranostic agents. Nano-bioengineering is still at a primitive stage of research as far as the diagnosis and treatment of SCLC and pleural mesothelioma are concerned. Some bio-nanocarriers that have been explored for treatment of the lung cancer sub-types like the SCLC and pleural mesothelioma are elaborated below.

Pleural mesothelioma is one of the most lethal forms of lung cancer characterized by poor prognosis, aggressive growth pattern and high relapse rates. It has often been debated for the lack of efficient diagnostic and therapeutic strategies. Affordable and specific biomarker-based diagnostic method for pleural mesothelioma has recently gained significant scientific attention. Advanced biotechnological techniques have identified potential exosomal family of ~570 proteins termed as “mesothelioma exosomal signature” that can serve the diagnostic purpose quite well [295].

Sakura et al. researched antitumor activities of hemagglutinating virus of Japan envelope (HJ-E) isolated from inactivated non-replicating Sendai virus. The HJ-E were observed to evoke direct antitumor immune response upon activation of natural killer cells, dendritic cells, and regulatory T-cell suppression through the RIG-I/ MAVS pathway upon their intratumoral administration. Phase I dose escalation tolerability, safety and primary efficiency studies were performed on the patients with chemoresistant malignant pleural mesothelioma upon the intratumoral and s.c administration of HJ-E. The results were affirmative in terms of prevention of relapse at the injected tumor site as well as its safety. Though no serious adverse effects were observed, transient fever and mild site-specific symptoms were observed [296].

Sakurai et al. devised a novel bio-nanocarrier composed of cationic liposome and hyaluronic acid lipid conjugate for pleural mesothelioma therapy. The novel bionanocarrier was synthesized through reductive amination process that allowed HA-terminal attachment to the cationic lipids of liposomes containing Cisplatin, while sparing carboxylate group. Upon intrapleural administration of these bio-nanocarriers at a dose of 1.5 mg kg^−1^ Cisplatin in an orthotopic mice model of pleural mesothelioma, the bio-nanocarriers demonstrated significantly higher CD44 expression and subsequently synergistic cellular uptake of Cisplatin in the mesothelioma cells [297].

Singh et al. report the development of a novel injectable/sprayable hydrogel composite with peptide-based microRNA nanoparticles to target pleural mesothelioma cells. The hydrogel serves as therapeutic depot for delivery of miRNA to the tumor cells. For the formation of stable nanoparticles, miRNA was firstly complexed with amphiphilic cationic peptide and these nanoparticles were incorporated into peptide hydrogel vai self-assembly of the amphiphilic cationic peptide to form a composite hydrogel. The formulation was optimized for properties like biopersistence, biostability, loading ability, specific targeting, bio adherenance and integrity. The miRNA nanoparticles were observed to be non-toxic and upon tracking of the Cy3-labelled miRNA, they demonstrated significantly higher clathrin receptor-mediated uptake. In vivo studies in orthotopic mice model demonstrated significantly enhanced anticancer activity, tumor regression, and higher overall survival [298].

Small cell lung cancer (SCLC) is second-most common type of lung cancer associated with very high mortality rates and poor prognosis (only 10% of the diagnosed patients survive for greater than five years).

Huang et al. developed a novel cytomembrane-mimicking bio-nanocarrier for targeted delivery of anticancer enzyme asparginase (ASP) to SCLC cells. The bio-nanocarriers are self-assembled structures composed of polyethylene glycol 2000 (HA-g-PEG) and α-cyclodextrin conjugated to endogeneous CD44 targeting component. The supramolecular assembly and vesicular encapsulation served as a bioreactor that facilitated loading of ASP in its hollow interior, protected the enzyme and provided targeted delivery. The bio-nanocarrier had explicitly higher stability, enzyme-substrate kinetics, catalytic activity and antitumor efficacy as compared to free ASP. The nanobiocarrier exhibited excellent in vivo safety and biocompatibility and can be employed for the treatment of several other malignancies [299].

Whitener et al. developed a unique DNA derivatized gold nanoparticles that can bind to ~1100 daunomycin molecules. The drug was held by the mutating nucleic acid strands of the bio-nanocarrier. The length and sequence of the double-stranded DNA of bio-nanocarrier can be modulated to enhance payload. Moreover, diverse array of aptamers can be used for facilitating binding of the nanocarrier to diverse targets. The highly specific, programmable bio-nanocarrier presents a highly specific, avidity driven nanosystem with high drug loading ability and biocompatibility administrable by intratracheal or intravenous route. The drug release rates and quantity can be modulated by mutating the base pairs of the drug-binding region and changing the sequence. The SCLC-specific aptamer-based bio-nanocarrier needs to be evaluated for *in vivo* efficacy in orthotopic nude mice model for ensuring safety and efficacy [300].

Bio-nanocarriers may also facilitate alteration in the route of administration (preferably inhalational, oral [301] over the conventional i.v / intratumoral or parenteral). This may increase patient compliance while avoiding the need for hospitalization [302–306]. Although not much explored until now, the concept may be extrapolated for the nano-biocarriers as well.

A few select examples from each of the therapeutic categories have been discussed in the respective sections.

#### Nano-biocarriers in Chemotherapy

Bacterial-derived minicells represent a breakthrough example of nano-biohybrids to have reached the clinical trial phase of approval. These 400-nm bio-nanocarriers have been claimed to offer targeted chemotherapy to many types of cancers including lung cancer. For facilitating tumor targeting, these minicells may be surface-functionalized using bispecific antibodies that bind specifically to both; the lipopolysaccharide component bacterial O-antigen as well as to the EGFR receptor. The versatile drug delivery carrier may be loaded with chemotherapeutics having different physicochemical properties (solubility, charge, and hydrophobicity) at therapeutic concentrations. The minicells were reported to incorporate vinblastine, cisplatin, and irinotecan that represent amphiphilic, hydrophobic, and hydrophilic prototypic cargo. The in vitro and in vivo evaluations in the A549 xenograft model provide compelling proof of the safety and efficacy of this drug delivery platform. Besides having an excellent antitumor activity (greater than 11 other antitumor treatments), these minicells are highly suitable for facilitating combination chemotherapy [307].

A novel nano-biohybrid drug delivery system, ‘nanofibrin’ was recently reported by Vedakumari et al. for the targeted delivery of erlotinib. The erlotinib-encapsulated nanofibrin was developed through the wet co-precipitation method and was subsequently conjugated to bevacizumab (anti-VEGF antibody) by employing the EDC/NHS method of conjugation. The bio-nanocarriers had a spherical shape with a surface positive charge and a hydrodynamic diameter of ~ 79 nm. The in vitro evaluations in the A549 lung cancer cell line proved the strategy to exhibit enhanced anticancer activity. Effective cell inhibition accompanied by an increase in the sub-G0/G1 phase with a subtle decrease in the G0/G1 phase was observed from the in vitro cytotoxicity assessment [168].

In another instance, Chen et al. developed hyaluronic acid (HA) and nitroimidazole (NZ) co-decorated polymeric (PNPs) and lipo-polymeric (LPNs) bio-nanocarriers for targeted delivery of cisplatin. While HA provided targeting to the CD 44 and CD 33 receptors that are overexpressed in lung cancer, NZ facilitated drug delivery to deeper hypoxic regions through bio-reductive prodrug designing and provided an amphiphilic drug delivery platform. The hydrodynamic particle size of LPNs and PNPs was observed to be 185.6 ± 4.7 nm and 136.7 ± 3.5 nm, respectively. In vitro cell uptake of the LPNs in the drug-resistant A549/DDP cell line was significantly greater than the PNPs, indicating the fusogenic property of the lipids to cell membranes. The LPNs and PNPs were observed to have remarkably higher antitumor activity as compared to the pure drug in drug-resistant human lung cancer A549/DDP cells. The in vivo survival *rates in* LNP-treated animal groups was higher (90%) as compared to the groups treated with PNPs (80%) vs the cisplatin-treated groups (30%) [308].

Novel biomimetic bio-nanocarriers composed of platelet cell membrane-derived were developed by Chi et al. for the targeted delivery of Docetaxel to the lung cancer cells. For the construction of these novel bio-nanocarriers, poly (lactide-coglycolide) (PLGA) nanoparticles, encapsulating DTX, were enveloped using the platelet membrane. The developed nano-biohybrid exhibited high drug loading capacity, sustained drug release, excellent stability, and tumor-targeting ability. The results of in vivo studies on A549 xenograft showed increased tumor regression accompanied by reduced adverse effects and toxicity [309]. Some other relevant examples are summarized in Table 2.

#### Nano-biocarriers in Phototherapy

**Fig. 5:**
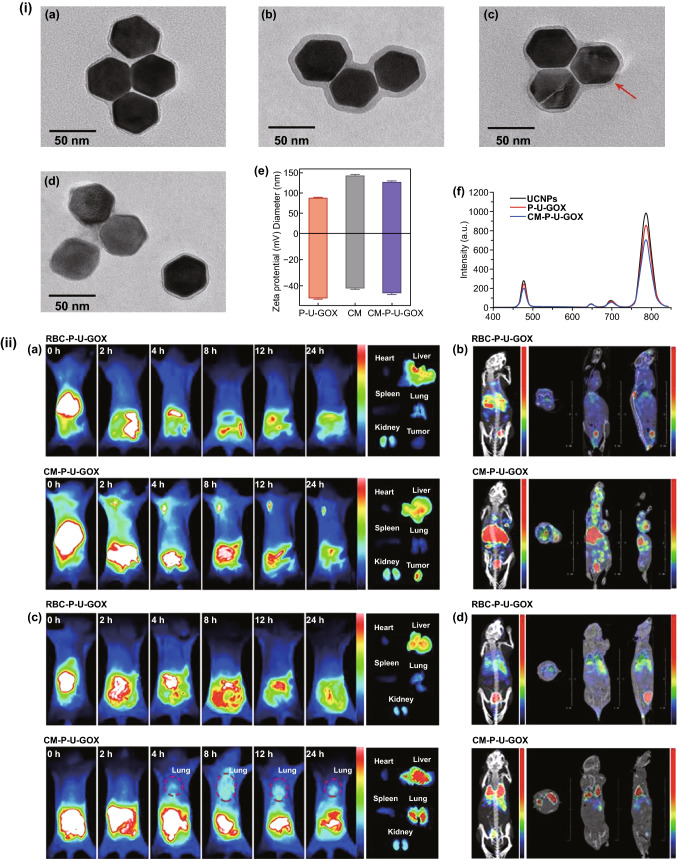
**(i)** TEM microphotographs of **a** PAAO-UCNPs-GOx, **b** CM-PAAO-UCNPs-2 GOx, **c** CM-PAAO3-UCNPs-GOx+ pH6.5 buffer solution for 30 min, **d** CM-PAAO-UCNPs-GOx + pH6.5 buffer solution for 2 h, **e** hydrodynamic particle sizes and electrokinetic potentials of PAAO-UCNPs-GOx, cell membrane, and CM-PAAO-UCNPs-GOx, **f** emission spectra of UCNPs, PAAO-UCNPs-GOx and CM-PAAO-UCNPs-GOx upon 980 nm irradiation. (**ii**) In vivo homotypic tumor targeting 4T1 tumor bearing mice after being administered ^125^I-CM-PAAO, UCNPs-GOx and ^125^I-RBC-PAAO-UCNPs-GOx. **a** fluorescence images, **b** nuclide images, **c** fluorescence images of 4T1-lung metastasis, and **d** nuclide images of 4T1-lung metastasis mice. Adapted from Ref. [310] with permission from Elsevier, Copyright 2020

Wang et al. have developed a novel nano-biohybrid for enhanced phototherapy of lung cancer. The research group developed a biomimetic nanoreactor with synergistic cascade starvation property and imaging ability at 980 nm radiation. The nanoreactor composed of polyacrylic acid-n-octylamine (PAAO) micelles-encapsulating NaYF4: Yb, Tm upconversion nanoparticles (UCNPs)-surface-modified using cancer cell membranes, i.e., (PAAO-UCNPs- GO_x_), was employed for homotypic cancer cell targeting. Cancer starvation was initiated through glucose oxidase (GO) catalysis that causes glucose breakdown, while the anticancer activity was promoted by the photolysis of hydrogen peroxide to hydroxyl radical. The PAAO-UCNPs- GO_x_ were observed to emit photons of 470 nm on being irradiated by 980 nm laser, which facilitated deep tissue photothermal therapy in the hypoxic tumor milieu. Furthermore, the fluorescent emission of 800 nm generated by the nanoreactor facilitated image-guided therapeutic monitoring. The in vitro and in vivo anticancer efficiency of this formulation was validated employing a 4T1 cell-induced metastatic lung cancer model (Fig. 5). The research findings affirm the developed biomimetic nanoreactor to be an efficient, smart, and simple approach for multimodal oncotherapy [310].

Yet another instance of the application of nano-biohybrid in phototherapy was available in the research work of Ouyang et al. They developed a nano-biohybrid composed of mesenchymal stem cell (MSCs) encapsulating the Chlorin e6 (Ce6)-conjugated polydopamine nanoparticles (PD-Ce6) for photodynamic (PD) and photothermal therapy (PT) of lung metastatic melanoma. The MSC-mediated phototherapy was facilitated by the ‘Trojan-horse’ like transit between the cancer cells and the MSCs, characterized by an interplay of endocytosis-exocytosis and re-endocytosis in addition to high tumor penetration and high tumor homing capability. The bio-nanocarriers exhibit no overt toxicity to the MSCs while being highly cytotoxic to the cancer cells. The highly selective cytotoxicity can be achieved after being triggered by NIR for inducing the PD/PT mechanisms. About 60% release of the phototherapeutic agents was observed after 72 h of MSC-PD-Ce6 bio-nanocarriers administration. In vivo evaluations in B16-F10 melanoma, cell-induced metastatic mice model displayed excellent anticancer activity, as apparent from the reduced number of metastatic colonies and survival rates in the animals treated with MSC-PD-Ce6 bio-nanocarriers as compared to the simple PDS-C6-based therapy [311].

Cao et al. developed a novel nano-biohybrid composed of the mesenchymal stem cells (MSCs) encapsulating the Ce6-conjugated manganese dioxide (MnO_2_) nanoparticles. MnO_2_ is an excellent oxygen-producing substrate, employed in the design of antitumoral nanomedicine to respond to hypoxic and acidic pH-stimuli. The MnO_2_-Ce6 were formed by physical adsorption of the Ce6 moiety to the MnO_2_ nanoparticles to increase the systemic circulation. The MSCs bound to the MnO_2_-Ce6 increased the cell uptake significantly while reducing the non-target toxicity. MnO_2_ released from the MnO_2_-Ce6 nano-biohybrid catalyzed the reactions that consume the abnormal cancer cell metabolic products like protons (H^+^) and hydrogen peroxide (H_2_O_2_)_._ Consequent formation of O_2_ resourced the formation of ^1^O_2_ upon being irradiated by 633 nm laser light, thereby incapacitating the hypoxic constraint of photodynamic therapy (PDT) for effective antitumor therapy. MnO_2_ decomposition to Mn^2+^ representing high T1 relaxivity in the MRI region, followed by its hepatic metabolism and renal excretion. The Mn^2+^ may greatly facilitate the direct MRI imaging of MSCs and tumor microenvironment (TME), providing a theranostic advantage. The in vivo evaluation in a xenograft mice model of LLC revealed higher survival rates, apoptosis, and tumor cell death in the animals receiving phototherapy with MSCs-MnO_2_-Ce6 as compared to the phototherapy with MnO_2_-Ce6 groups, indicating high anticancer activity. The MSCs-MnO_2_-Ce6 besides being highly biocompatible and biodegradable had no significant systemic toxicity. Some other relevant examples from this category of anticancer activity are discussed in Table 3 [312].

#### Nano-biocarriers in Immunotherapy

Chiang et al. formulated a fucoidan-based magnetic nano-biohybrid for tumor-targeted immunotherapy of checkpoint inhibitors. Despite the remarkable clinical success, anti-PD1 inhibitors are ineffective in a subset of patients with compromised immunity and have immunotoxicity constraints. Novel bio-nanocarriers composed of magnetic nanoparticles and fucoidan-dextran (IO- FuDex3) conjugated to T-cell activators (anti-CD28 and anti-CD3) or checkpoint inhibitors (anti-PD-L1) were developed and evaluated for their efficacy in lung cancer. The IO-FuDex3-nano-biohybrid had an immuno-restorative effect in the immunocompromised patients, mediated by tumor-infiltrating lymphocytes, and tumor targeting was achieved using the external magnetic drive. The nano-biohybrid-based immunotherapy (at dose < 1%) was observed to extend the median survival of animals from 32 to 63 days, with low systemic toxicity in a 4T1-metastatic model of lung carcinoma [313].

Granzyme B is a natural serine protease, secreted by the natural killer cells and cytotoxic T cells. Despite being a highly effective anticancer agent, such peptides lack biostability and have low cellular permeability, which desists their therapeutic effectiveness. Yang et al. developed reduction-responsive polymerosomes loaded with Granzyme B for immunotherapy of lung cancer. The polymerosomes were surface-functionalized with cell-penetrating peptides to facilitate targeted intracellular delivery of the enzyme. The polymerosomes were reported to have a particle size of 82–90 nm, reduction-sensitive drug release property, high cellular internalization, and cytoplasm-specific protein release in the A549 cells. The FITC-labeled cytochrome C-mediated model protein analysis of the developed polymerosomes depicted protein loading of ~ 17.2%w/w. The cell line studies of the polymerosomes on A549 cells demonstrated high anticancer activity (IC50 of 20.7 nM) and high cell-penetrating ability. In vivo evaluation in orthotopic A549-Luc pulmonary tumor-bearing nude murine model demonstrated tumor inhibition in the treatment groups receiving granzyme B-polymerosomes at a dose equivalent to 2.88 nmol kg^−1^ GrB. This was accompanied by significantly increase survival rates over the non-targeted and untreated disease control groups, suggesting this approach to be an effective immunotherapeutic modality.

Immuno-liposomes are a well-known class of nano-biohybrid with multiple oncological applications. Repic et al. developed immunoliposomes, with antibody fragment (Fab) targeted to lung cancer-specific antigen. While covalent coupling is used for the conventional conjugation method of antibody fragments, these novel immunoliposomes were formulated employing a novel formulation strategy that obviated the use of cross-linking agents. For precise conversion of the MAbs to recombinant antibody fragments, P. pastoris. P. pastoris were employed as hosts. The C-terminal of the recombinant Fab antibody fragment was fused to a peptide derived from pulmonary surfactant protein D and incorporated into the liposomal bilayer to fabricate the immunoliposome. The immunoliposomes containing the CD59 and CD48 FAbs were thus observed to have dual specificity. The Fab-labelled immunoliposomes were demonstrated to target the antigen-positive cells with great affinity and specificity [314]. The multimodal platform can be employed for the targeted delivery of several therapeutic and imaging agents to the cells overexpressing the receptors for these antibodies, like lung cancer [315]. Some other examples of nano-biohybrid-based immunotherapeutics that have been applied in lung cancer are discussed in Table 3.

#### Nano-biocarriers in Gene/Epigenetic Therapy

Numerous bio-nanocarriers have been researched extensively for facilitating gene delivery. It has been well established that the replenishment of the downregulated miR-660 gene has the potential to inhibit the proliferation of lung cancer cells through the restoration of the MDM2-P53 axis. However, the administration of the miRNA has been a challenging task owing to the poor in vivo bioavailability issues. Moro et al. formulated bio-nanocarriers composed of cationic lipid 1, 2-biology-1-3- tri-methylammonium propane (DOTAP) to facilitate targeted delivery of miR-660 gene to lung cancer cells while increasing its bioavailability. In vivo experiments, tumorigenesis was induced in the SCID mice using P53 wild-type patient-derived xenografts (PdX). Upon biweekly administration at a dose of (1.5 mg kg^−1^) for four weeks, a significant reduction in tumor growth was observed amongst the SCID mice expressing two different P53 with no systemic toxicity. Significant inhibition of the MDM2 and restoration of the P53-downstream signaling pathway were observed in the animal groups receiving the cationic liposomal formulation, suggesting potential anticancer activity. Moreover, the metastatic potential of the H-460 cell was reported to be greatly disrupted by stable bioexpression of the miRNA cells [316].

The inhalational route of administration has gained incredible attention from the researchers to facilitate the localized treatment of numerous morbidities including lung cancer. However, due to the challenges associated with the inhalational delivery of the genes, the search for novel vectors that can effectively and safely deliver the genetic cargo has not ended [317]. Also, targeted delivery to the lung is a challenging task owing to the high perfusion rate, which disfavors the localization of the nanoparticles therein. To address these issues, Yunfeng Yan developed polyester bio-nanocarriers for aerosolized delivery of siRNA to lung tumors. Out of 540 members scrutinized from the functionalized polyester library, PE4K-A13-0.33C6 and PE4K-A13-0.33C10 were found suitable for the delivery of siLUC-targeted siRNA to the lungs, through the formation of stable nano-biohybrid, ‘polyplexes’. The serum stability of these polyplexes was improved by employing Pluronic F-127 or PEG 2000 DMG to reduce their surface charge. The polyplexes were reported to be endocytosed by the A549-Luc cells within 4 h of administration. Upon aerosolized delivery in mice with orthotopic lung tumors, the polyplexes were observed to undergo selective biodistribution and targeting, resulting in effective gene silencing in the tumor cells [318].

Elwakil et al. developed bio-nanocarriers with GALA-peptide-functionalized nanoparticles composed of pH-sensitive lipids for delivering siRNA that prevents metastatic dissemination of lung cancer. Besides serving as a lipid matrix, the pH-sensitive lipid (YSK05) also effectively caused gene silencing, and GALA (a peptide with 31 amino acids) facilitated the endosomal escape. The developed nano-biocarrier was found to be 40-fold more efficient in inducing gene silencing and higher anti-metastatic potential than the formerly developed membrane-based nanodevice. Effective gene silencing was observed at 0.01 mg siRNA/kg dose, with no reports of other non-targeted toxicity [319].

**Fig. 6:**
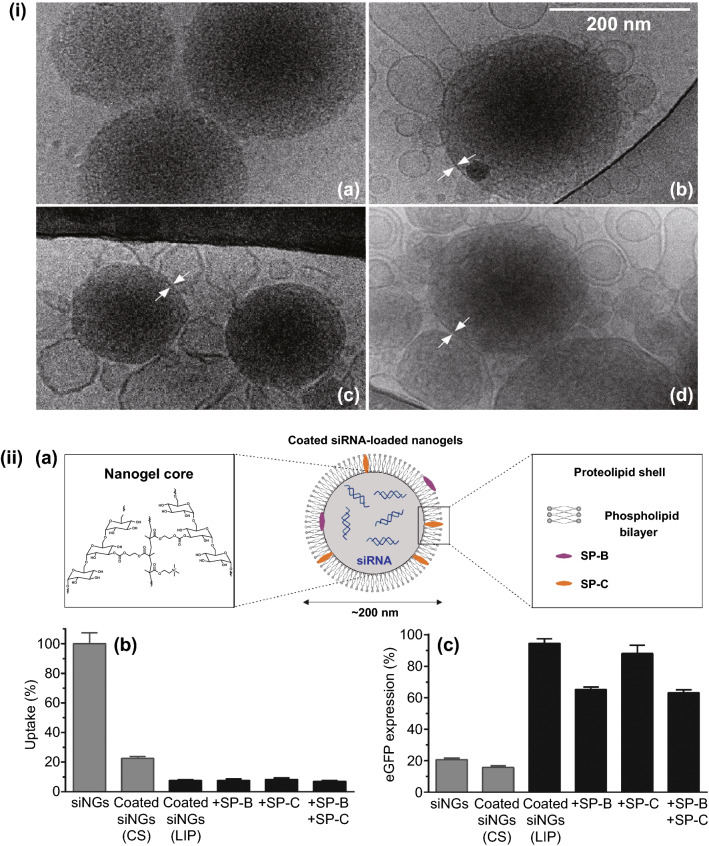
**(i)** Cryo-TEM microimages of different nanoformulations: **a** uncoated, **b** Curosurf® coated. **c** DPPC:eggPG:SP-B-coated, and **d** DOPC:eggPG:SP-B-coated siRNA-loaded nanogels (siNGs). **(ii)** Proteolipid shell over the siNG core is depicted by white arrows. **a** Illustration of (proteo)lipid-coated siRNA-loaded nanogels (siNGs), **b** comparative cell uptake, and **c** gene silencing capacity of (siNGs) in H1299_eGFP cells determined employing flow cytometry. The siNGs were layered using DPPC:POPC:eggPG (50:35:15 wt%; the coated siNGs (LIP) and Curosurf® (coated siNGs (CS)) or in this LIP outer layer, SP-B (0.4 wt%) and/or SP-C (0.7 wt%) was incorporated. For cell uptake studies, the siRNA (siCy5) was tagged with fluorescent dye and their formulations wherein significant eGFP-targeting siRNA (siEGFP) normalization was observed as compared to EGF expression in animals treated with control siRNA (siCTRL). Adapted with permission from Ref. [320] with permission from Elsevier, Copyright 2018

The research work by Merckx et al. exemplifies how the endogenous surfactant protein b may augment the inhalational delivery of siRNA-encapsulated proteolipid-layered nanogels. Led by the promising results obtained with bio-nanocarriers composed of the Curosurf®, the researchers identified major constitutional components contributing to an improvement in the particle biostability and facilitating in vitro and in vivo siRNA delivery. Wherein, surfactant protein B (SPB) reconstituted in proteolipid-coated nanocomposite/nanogels was identified to be one of the most potent delivery aid (Fig. 6). An effective anticancer activity with no systemic cytotoxicity was observed on their in vitro and in vivo evaluations. Cancer-associated macrophages can also be targeted by employing this novel nano-biohybrid, presenting a novel and extremely useful tool for effective targeting to lung cancer via the inhalational route of administration [320].

#### Nano-biocarriers in Radiotherapy

Radiation therapy is one of the most important oncology modalities. In patients with radio-resistant oncological cell populations, various nanoparticle-mediated radiosensitization strategies have materialized to be a useful therapeutic aid [321].

**Fig. 7:**
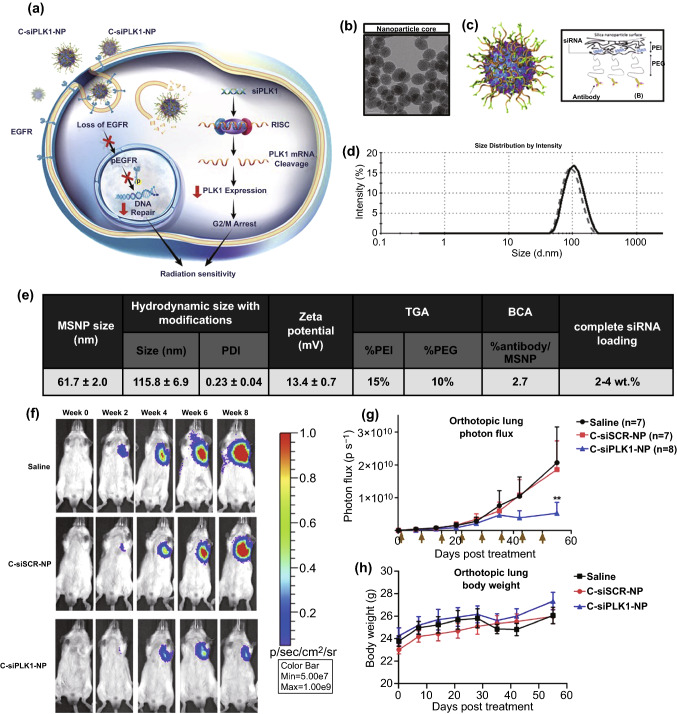
Cetuximab-conjugated mesoporous silica nanoparticles (MNP) loaded with PLK1 siRNA (siPLK1) or C-siPLK1 for EGFR-targeted delivery. **a** radiosensitization strategy with combined EGFR antibody and siPLK1-nanoparticles. The C-siPLK1-NPs bind to EGFR receptors and are endocytosed to result in loss of EGFR and phosphorylated EGFR to initiate DNA repair, while reducing DNA damage due to radiation and releasing the siPLK1 to cytosol. The siPLK1 reduces the PLK1 protein expression and arrests cell arrest at G2/M, thereby sensitizing the cells to radiation damage. **b** TEM microscopic images of 50-nm MNPs. **c** Layer-by-layer surface-conjugated nanoparticles. **d** Hydrodynamic diameter of C-NP (solid lines) and without siRNA(dotted lines). **e** Particle size, zeta potential and other characteristics like drug loading and antibody conjugation of C-siRNA-NPs expressed as mean ± SD. **f** IVIS imaging in orthotopic lung tumor-induced mice after being treated with C-siPLK1-NP, C-siSCR-NP, or saline. **g** Tumor growth determined using photon flux in prone and supine position of the treated mice (*n* = 7–8). Arrows indicating days of treatment. (H) Body weight of mice post-therapy. Adapted from Ref. [322] with permission from Elsevier, Copyright 2019

While PLK1 inhibition has been well established to sensitize the cancer cells to radiation via mitotic regulation, EGFR is reported to be an arbitrator of DNA repair and is upregulated in ~ 50% of pulmonary carcinoma patients. Based on these facts, Reda et al. developed a novel nano-biohybrid for radiosensitization of the non-small cell lung cancer cells (NSCLC). For optimizing the synergy of radiotherapy and targeted delivery, the research group devised cetuximab-conjugated nanoparticles, encapsulating siRNA-directed against polo-like kinase 1 (PLK1)**.** Thus, formulated bio-nanocarriers (C@siPLK1-NH) were reported to effectively target the EFGR-positive A549 cells, causing apoptosis by G2/M cell cycle arrest. The C@siPLK1-NH were observed to have excellent translational potential in an orthotopic murine model of lung cancer, which manifested as reduced tumor proliferation and prolonged survival rates (Fig. 7). Similar anticancer synergy with radiation therapy was observed in breast cancer and colorectal cancer also [322].

In another instance, Travis et al. developed a nano-biohybrid of gold nanoparticles, surface-modified with cilengitide (a linear/cyclic RGDfV pentapeptide sequence targeted to αvβ3 integrin receptor) to cause radiation sensitization. The radiosensitization capacity of developed nanoparticles and the 137Cs irradiation was evaluated by clonogenic assay on αvβ3-overexpressing tumor cells like H460, HUVEC, and MCF7. The developed nano-biohybrid was observed to induce the dose enhancement factors (DFs) significantly, which was insignificant in the treatment groups receiving radiation therapy alone and the groups treated with radiation and unconjugated gold nanoparticles. Moreover, it was concluded from the observations of the DFs that cyclized RGDfV pentapeptides had higher radiosensitization capability as compared to its linear counterpart and the radiosensitization efficacy bio-nanocarriers was almost equivalent to the radiation therapy with cilengitide [323].

#### Nano-biocarriers in Combination Therapy

Combination oncotherapy has recently emerged as one of the most feasible therapeutic approaches to tackle complex diseases like cancer. A combination of different therapeutic modalities has led to a paradigm shift in oncology [292].

A unique therapeutic approach, combining the targeting ability of natural cancer cell membrane with the photothermal property of plasmonic gold nanorods and cytotoxic effect of β‑Lapachone (β-LN), was adopted by Marangoni et al. for the treatment of lung cancer. β-LN is a pro-drug like an anticancer agent that is activated by NADP(H): quinine oxidoreductase enzyme, that is intrinsically produced by the cancer cells of solid tumors like lung cancer. Plasmonic nanorods have been established to exhibit excellent photothermal activity upon IR irradiation. This research was undertaken to assemble the advantages of both these therapeutic modalities by fabrication of a multifunctional nano-biohybrid, composed of lung cancer cell (A549)-derived membrane co-loaded with PEGylated plasmonic gold nanorods and β-LN. The system exhibited NIR-triggered release of both the payloads from the cancer cell membrane vesicle. The developed nano-biohybrid demonstrated high in vitro cytotoxicity. In vivo histopathological examination of tumors in cancer-induced animals treated with the nanohybrid revealed excellent antineoplastic potential as compared to the groups receiving only radiation therapy [324].

Bio-nanocarriers combining chemotherapy and gene therapy were developed by Li et al. to combine the anti-angiogenic activity of vascular endothelial growth factor (VEGF)-siRNA with anticancer activity of etoposide (ET) for the treatment of metastatic and malignant forms of lung cancer. The nano-biohybrid constructs are designed using the cationic liposomes co-loaded with (VEGF)-siRNA and ET, enveloped with PEGylated histidine-grafted chitosan-lipoic acid polymer. The developed nano-biohybrid exhibited an excellent tumor penetration and high cellular endocytosis in addition to the pH-triggered release of the siVEGF and ET in an orthotopic mice model of NSCLC. The anticancer and antimetastatic potential of the nano-biohybrid is clear from the in vivo data that supports its high tumor growth suppression and antimetastatic potential [325].

Song et al. have developed bio-nanocarriers to facilitate combination therapy (photodynamic therapy and immunotherapy) for the effective treatment of metastatic lung cancer. Photosensitizer protoporphyrin IX (PIX) was conjugated to a chimeric peptide C16-K(PpIX)-PEG8-KDEVD-1MT (MT) (immune checkpoint inhibitor) via caspase-responsive peptide Asp-Glu-Val-Asp for developing nanoparticles by self-assembling upon dispersion in PBS. The nano-biohybrid thus developed had a high EPR effect and potent antitumor activity. The antitumor activity was mediated by ROS production at 630 nm on NIR irradiation and caspase-3-mediated intratumoral release of MT, thereby evoking an intense CD8+ T cell-induced immune response in lung cancer cells. The strategy was reported to inhibit growth as well as metastasis of lung cancer cells and can be foreseen as a potential clinical-oncotherapy candidate [326].

#### Novel Nano-biocarrier-Based Anticancer Therapy

Some recent and noteworthy examples of bio-nanocarriers, used for targeted delivery of novel categories of anticancer agents to lung cancer, have been discussed in this section. Liu et al. developed graphene oxide (GNO) nanocomposites functionalized with polysaccharide for targeted delivery of a novel anticancer agent SNX-2112 (Hsp90 inhibitor) to lung tumors. The unique physicochemical characteristics of the GNO make them an attractive material for constructing nanocarriers. Surface modification of the GNO-based nanocomposites with polysaccharides like chitosan (CH) and hyaluronic acid (HA) greatly improves its biocompatibility, while targeting the payload to the CD44 positive tumor cells (GNO@CH-HA). SNX-2112 was thereafter loaded in the GNO@CH-HA. SNX-2112 was released at a higher amount and release rate in acidic tumor conditions, conferring an additional TME-targeted property. While being highly cytotoxic to A549 cells, the SNX-2112-loaded GNO@CH-HA nanocomposites showed no cytotoxicity on erythrocytes or normal human bronchial epithelial cells. In vivo toxicity evaluation in Sprague–Dawley rats showed a short-term inflammatory response, but no long-term injury was witnessed. The novel nanocomposite was concluded to be a safe and effective means of targeted onco-therapy [327].

**Fig. 8:**
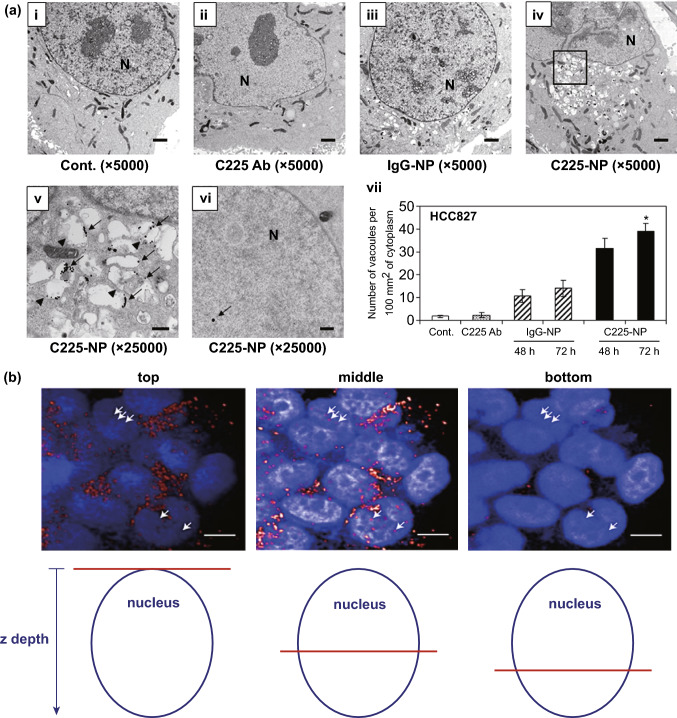
**a** In situ localization of C225-NP in the NSCLC cells. Electron micrographs presenting the ultrastructure of a cell, including the nucleus (n), of HCC827 cells treated with 0.065 mg/ml of C225 antibody, IgG-NP, or 0.661010 particles of C225-NP for 72 h. (i) Non-treated cells, (ii) C225 antibody-treated cells, (iii) IgG-NP-treated cells, (iv) C225-NP-treated cells, (v) a magnified view of the area boxed in (iv). The arrow indicates C225-NP, and the arrowhead indicates autophagosomes of including residual material and NP in the cytosol and C225-NP inside the nucleus. Arrow indicates NP in the nucleus. Scale bar = 1 mm. (vii) number of vacuoles per μm^3^ of cytoplasm after 48 and 72 h *P value, 0.05 vs control, C225 antibody and IgG-NP. **b** Confocal microscopy images of C225-NPs localized in the nucleus (top panel), where nuclei have been stained with DAPI dye and C225-NP localization can be marked in red. A specific area of *xy* plane of the nucleus at locations above or below the nucleus is denoted by the arrows indicating middle, top and bottom, respectively, whereas the Z-position of image within the nucleus is indicated by the sketch in the lower panel, wherein the red horizontal line represents the depth within nucleus from where the cross sectioning was made, Scale bar = 10 μm. Adapted from Ref. [328] under the terms of Creative Commons Attribution License as permitted by PLoS One, Copyright 2011

In another instance, Yokoyama et al. developed a nano-biohybrid targeted to EGFR for synergistic anticancer activity through induction of apoptosis and autophagy. To develop a highly targeted formulation, plasmonic magnetic nanoparticles (PMNs) were surface-functionalized with anti-EGFR antibody (Clone 225) that target the NSCLC cells with great selectivity (Fig. 8). Thus, developed C225-PMNs were observed to inhibit the EGFR-transduction pathway and high cytotoxicity in the EGFR-positive cells, while not affecting EGFR-negative cells [328]. Other few examples that can be considered under this category are discussed in Table 3.

Despite promising preclinical results, the clinical transformation of these formulations remains questionable due to the challenges associated with their active targeting ability. Due to large hydrodynamic sizes, multi-functionality, non-specific immune interactions, ‘on target but off the site’ response, low bio-stability and difficult nanoparticle conjugation, the efficacy of antibodies and proteins for functionalization is dubious. While the antibody fragments and peptides reduce the size to facilitate their conjugation to the surface of the nanoparticles, their efficacy is challenged by nonspecific biological interactions and peptide geometry. Aptamers have low circulation stability and are prone to degradation by nucleases that are non-specifically attached over the surface of nanoparticles. Small molecules, despite being effective, have a high cost for personalization and targeting. Receptor-negative cell population, genetic mutations, and high inter- and intra-patient diversity play an important role in determining their effectiveness. Moreover, most of the above-stated approaches may be unfeasible to be devised with precision at a large scale [101]. Employing bionanotechnology-based approaches to devise natural and semi-synthetic bioinspired/bioderived bio-nanocarriers may help overcome the majority of these challenges while reinforcing their targeting ability [15]. However, the novel approach is still under reappraisal and demands extensive efforts to bring the concept to reality.

### Nanobiotheranostics in Lung Cancer

The term ‘nanobiotheranostics’ refers to a unified diagnostic and therapeutic application of a single agent. Theranostics not only facilitates concurrent diagnostic and therapeutic aspects but also facilitates image-guided drug delivery and therapeutic drug monitoring. While nanotechnology has significantly contributed to advance this field, bionanotechnology-derived tools are being explored to overcome the limitations of the former. The majority of the nano-biotheranostics that have been employed till now include the inorganic nanoparticles, nanocages, nanoshells, nanoclusters, and metallo-organic frameworks composed of gold, silver, iron nanoparticles, silica, liposomes, dendrimers, micelles, and other bio-polymers conjugated to the bio-molecular ligands. However, with advancing bionanotechnology, several bioinspired bio-nanocarriers such as nanobots and cell-derived nano-biocarriers have emerged [4]. Several virus-based, protein-based, and apoferritin nano-biocarriers that have been explored for their theranostic applications in lung cancer have been discussed elsewhere [348]. Some of the recently explored approaches that have an immense potential to bring breakthrough in lung cancer theranostics have been discussed here.

The research work by Shevtsov et al. is a unique theranostic approach that combines the immunotherapeutic ability of Granzyme B (Gn B) with the diagnostic ability of superparamagnetic iron oxide nanoparticles (SPIONs) to target many types of cancers including lung cancer. The surface-modified SPIONs target a membrane-bound protein, Hsp 70, that is selectively overexpressed over the tumor cells but is absent over the normal cells. Gn B is a serine protease that is produced by the immune cells like the NK and T cells, that selectively targets the HsP70 receptors. The GnB-functionalized SPIONs (GnB-SPIONs) were reported to undergo selective endocytosis into the tumor cells and provide significantly higher MRI contrast and apoptosis-inducing ability. The therapeutic efficiency of GnB-SPIONs was observed to increase still further by employing a regimen combining magnetic targeting and stereotactic radiotherapy [349].

Yet another theranostic approach was employed by Duman et al. to facilitate tumor targeting to EGFR-positive cancer cells like lung cancer cells. For facilitating tumor targeting, the research group developed cetuximab-conjugated Ag_2_S quantum dots entrapping 5-fluorouracil (5-FU). The theranostic quantum dots were observed to overcome the issues of drug resistance and pro-survival mechanisms in A549 cells while facilitating targeted NIR imaging. The novel nanohybrid was observed to induce significant apoptosis in EGFR-positive lung cancer cells by suppression of autophagic mechanisms [350].

Cheng et al. developed a nano-biohybrid composed of Ag_2_S quantum dots (QD), entrapped in the erythrocyte membrane for combining the benefits of non-invasive sonodynamic therapy with imaging ability. The novel approach employs Ag_2_S quantum dots for sonosensitization while utilizing the endogenous erythrocytic catalase-mediated O_2_ as sonodynamic therapeutic. This is the first instance of employing sonodynamic therapy as an oncological modality, which is difficult to be employed otherwise due to hypoxic tumor conditions. To overcome challenges associated with spatial tumor characteristics, the research groups utilized a novel theranostic formulation that had an intrinsic source for O_2_ production. To increase the sensitivity of sonodynamic therapy through increased intracellular O_2_ production, phenethyl isothiocyanate (PEITC) was administered orally. The theranostic agent can be image-guided to the tumor tissue by employing fluorescence-NIR II-aided imaging technique and subsequently subjected to ultrasound to evoke reactive oxygen species (ROS) generation and cause subsequent cell death. The in vivo evaluation of the nano-biohybrid demonstrated long systemic circulation, exceptional biocompatibility accompanied by high anticancer efficacy in a xenograft mice model [351].

c-MET (hepatocyte growth factor receptor) aberrations have been well reported in many forms of cancers including lung cancer and metastatic malignancies. Lu et al. have developed a novel nano-biohybrid employing anti-*c-Met* human single-chain variable fragment (scFv) antibody as a vector. The anti-c-Met scFv fragment was attached over the surface of PEGylated liposomes encapsulating doxorubicin. The nano-biohybrid offered excellent targetability while facilitating in vivo fluorescent imaging and tumoral cell uptake as apparent from the tumor to normal ratio. Thus, devised immunoliposomes demonstrated high antitumor activity in the H460 xenograft mice model, suggesting its potential as a theranostic agent [179].

An innovative approach for theranostic relegation of progression and metastasis of lung adenocarcinoma has been reported by Chan et al. The authors of this research work have developed novel nanomaterial by employing silica shell-based nanocarrier, which serves as near infrared-persistent luminescence nanoparticles (NIR PNs). The NIR PNs were optimized for their physicochemical properties to attain a prolonged NIR luminescence. For an additional therapeutic effect, afatinib (AT) was conjugated to the PNs, and was subsequently functionalized with MAGE-A3 (Map) to obtain C). MAGE-A3 is a DNA aptamer, targeted against tumor-specific melanoma-associated peptide antigen (MAGE-A3111-125), that is overexpressed in lung cancer and many metastatic forms of cancer. Due to the advantages like highly modifiable structure and specific/targeted tumor targeting, this nano-biohybrid may be envisioned as a future of image-guided theranostics [352].

## Contemporary Erudition and Outlook

With several positive outcomes, the application of doctrines of nano-bioscience and bio-nanotechnology has presented an immense potential to redefine the current oncological scenario. An entire armada of different forms of bio-modified nanocarriers is being researched to subjugate various hurdles in the way of oncotargeting. Nanobiomedicines, by the virtue of their three-dimensional, multicomponent array exhibit certain distinct colloidal characteristics that bear an immense potential to transfigure the current state of oncotherapy. These future ‘nano’ tools have been supposed to cast their idealism upon the forthcoming preventive, diagnostic, therapeutic as well as theranostic aspects of several forms of neoplasms including lung cancer. Various novel nanosystems have been extensively explored to solve various issues confronted by conventional therapies like poor tumor bioavailability, nonspecific drug targeting, drug resistance, off-target side effects as well as detection of cancer cell progression and metastasis. However, the prognostic significance of nanotechnology and biotechnology in the oncological context is constrained and demands remarkable improvement.

An entirely different perspective on oncology awaits unanimous efforts in the field of nano-biosciences and bio-nanotechnology. Bio-nanotechnology tools may help envision and discover several novel oncological aspects by providing kaleidoscopic vision through molecular diagnosis and advanced bioimaging techniques. Several novel targets may be identified and novel therapeutic strategies be devised for reinforcement of oncotherapy. Revisiting the principles of nano-biosciences may help leverage the oncological sciences to a considerable extent by devising novel strategies that smoothen the trajectory to target the tumors. Nano-biocarriers may be explicitly designed to assist the delivery of various types of cargoes to the tumor tissue while overcoming various physicochemical and biological barriers. Additionally, the nano-biocarriers are a useful means for addressing tumor heterogeneity through personalized and tailored-drug delivery. The concept of combination therapy for targeting as well as theranostic purpose may also be facilitated using several forms of nano-biocarriers. Bionanotechnology-derived theranostic and other multi-functional arsenal has an immense potential to empower the oncology and expand the horizon toward optimistic outcomes.

However, the factual translation of nanomedicine may be made possible only by overpowering the challenges associated with them. The major challenges faced by bionanotechnology-based approaches are related to the lack of comprehensive interdisciplinary understanding amongst researchers of different scientific disciplines. Some of the other challenges associated with the use of nanomedicines relate to their biological repercussions, pharmacoeconomic impact, scale-up, immunological and toxicological concerns due to inter-patient bio-variance and modulated pharmacokinetics and biodistribution. Subtle alterations in the pharmacokinetic behavior of nanoparticles may often impede the in vivo biodistribution and alter the tumor bioavailability of the drug, thereby reflecting their impact on its pharmacodynamics. Bio-phenomenon like nano-biointeractions, bio-molecular corona formation, RBC hitchhiking, hepatic sequestration of nanomedicine for prevention of metastasis, etc., that have been less explored also needs to be extensively researched to deduce the complete therapeutic serendipity of the nano-biocarriers [353]. Björnmalm et al. have provided a concise overview of several approaches employed for bridging the bio-nanosciences and cancer nanomedicines in their review [353].

Streamlined efforts of the researchers from mathematical, engineering, chemistry, physics, and biology disciplines may help devise models for predictive analysis of the raw research data and findings gathered through bionanotechnology. Besides this, regulatory guidelines on the validation, functionalization, processing, safety, and toxicity assessments of nano-biocarriers and nano-biodevices also need to be devised. While there is a long way to safeguard the clinical applicability of bionanotechnology-derived tools, pacing up data integration and facilitating applications of validated predictive analytical and biomodeling tools may significantly pace up the process of their regulatory approval. With compelling shreds of evidence from current advances in the bionanotechnology-derived tools, their application in oncology proclaims a promising futuristic scenario.
